# Supplementary material for: Fine-scale population structure and widespread conservation of genetic effect sizes between human groups across traits
Source: Nat Genet. 2025 Feb 3;57(2):379–89. doi: 10.1038/s41588-024-02035-8 (PMC11821542; doi:10.1038/s41588-024-02035-8)
Supplement: Supplementary file 1 — Supplementary table legends, figures and Notes 1 and 2. [file 41588_2024_2035_MOESM1_ESM.pdf]

nature genetics

Supplementary information

https://doi.org/10.1038/s41588-024-02035-8

Fine-scale population structure and widespread conservation of genetic effect sizes between human groups across traits

In the format provided by the authors and unedited

Table of Contents

Legends of supplementary tables

Supplementary Figures

Supplementary Note 1

Supplementary Note 2

Legends of supplementary tables

Supplementary Table 1. 127 UK/Ireland and world-wide ancestries, and their abbreviations, as identified and used in the ancestry pipeline.

Supplementary Table 2. Mean ancestries in each birth place region/countries from each of the 127 ancestry groups that genetic similarity is compared to a). birth place region. b). birth place countries.

Supplementary Table 3. Properties of SNPs showing genome wide significance in AC-corrected GWAS (subtable labels) vs only in PC-corrected GWAS (alternate subtables) for two regionally defined traits: Birth place (North/South) and Employment score (England). Only those SNPs showing prior GWAS association evidence, for the traits catalogued in Table S6, are listed (this removes many SNPs in particular significant only in the PC-corrected GWAS). Note that the majority of SNPs significant after AC-correction for employment score show prior GWAS evidence for traits related to cognitive function, educational attainment, or socioeconomic status, while few PC-only-associated SNPs show prior GWAS evidence. a) Independent SNPs significant in AC-corrected GWAS for Birth place (North/South). b) (PC-only) independent SNPs significant in PC-corrected GWAS for Birth place (North/South). c) Independent SNPs significant in AC-corrected GWAS for Employment score (England). d) (PC-only) independent SNPs significant in PC-corrected GWAS for Employment score (England). e) Independent SNPS significant in only the AC-corrected GWAS, for waist circumference.

Supplementary Table 4. Counts of independent hits in three traits: Birth place (North/South), Employment score (England) and Waist circumference, as shown in main Fig. 3c-e. For each trait, the counts are stratified by three categories: “AC-specific”, ”AC, PC shared” and “PC-specific”. Within each category for each trait, we count the number of independent hits overlapping traits of interest, which is defined in the second row, and count the number of independent hits with NO GWAS evidence. For Birth place (North/South) and Employment score, we provide odds and p-value for trait of interest for AC-hits versus non-AC hits, and for

no GWAS for AC-hits versus non-AC hits. P-value is obtained by χ^2 test with 1 degree of freedom.

Supplementary Table 5. Results of running a joint regression to investigate correlations with PCs, for those SNPs in Table S2 associated with waist circumference after AC-correction but not PC-correction. Each sheet indicates the results for regression of the genotype of the labelled SNP versus the first 20 PCs; note that PC20 shows a strong and dominant correlation in each case, corresponding to the high loadings of this PC in this region of the genome (Figure S9) and indicating this PC might result in false negatives. a) SNP chr15:84311431_TA_T. b) SNP rs28363946. c) SNP rs4843158. d) SNP rs4125566. e) SNP rs35641144.

Supplementary Table 6. Correlations of (centralised) PGS constructed by AC-corrected and PC-corrected GWAS for 7 ancestry groups across 53 traits.

Supplementary Table 7. Data sources for constructing the ancestry "painting" reference panel.

Supplementary Table 8. Demographic and phenotypic UKB fields used in the AC-corrected and PC-correct GWAS.

Supplementary Table 9. Queried traits for GWAS hits in AC and/or PC in three UKB phenotypes: "Birth place (north/south)", "Employment score" and "Waist circumference", and the phenotype categories into which they were assigned.

Supplementary Figures

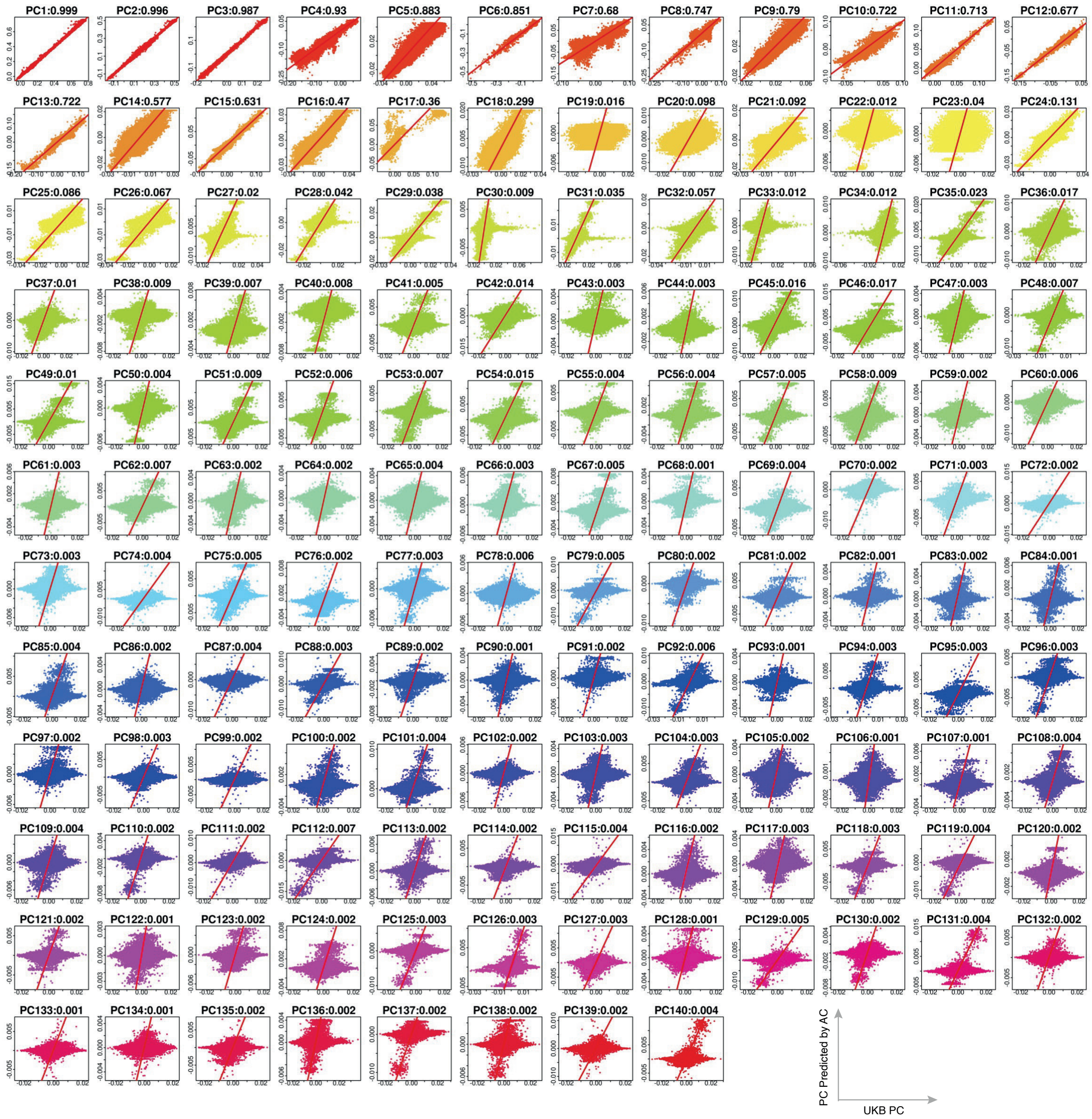

Supplementary Fig. 1. Using 127 ACs to predict 140 PCs across all UKB participants. The X-axis shows observed PCs, and the y-axis shows AC-predicted PCs.

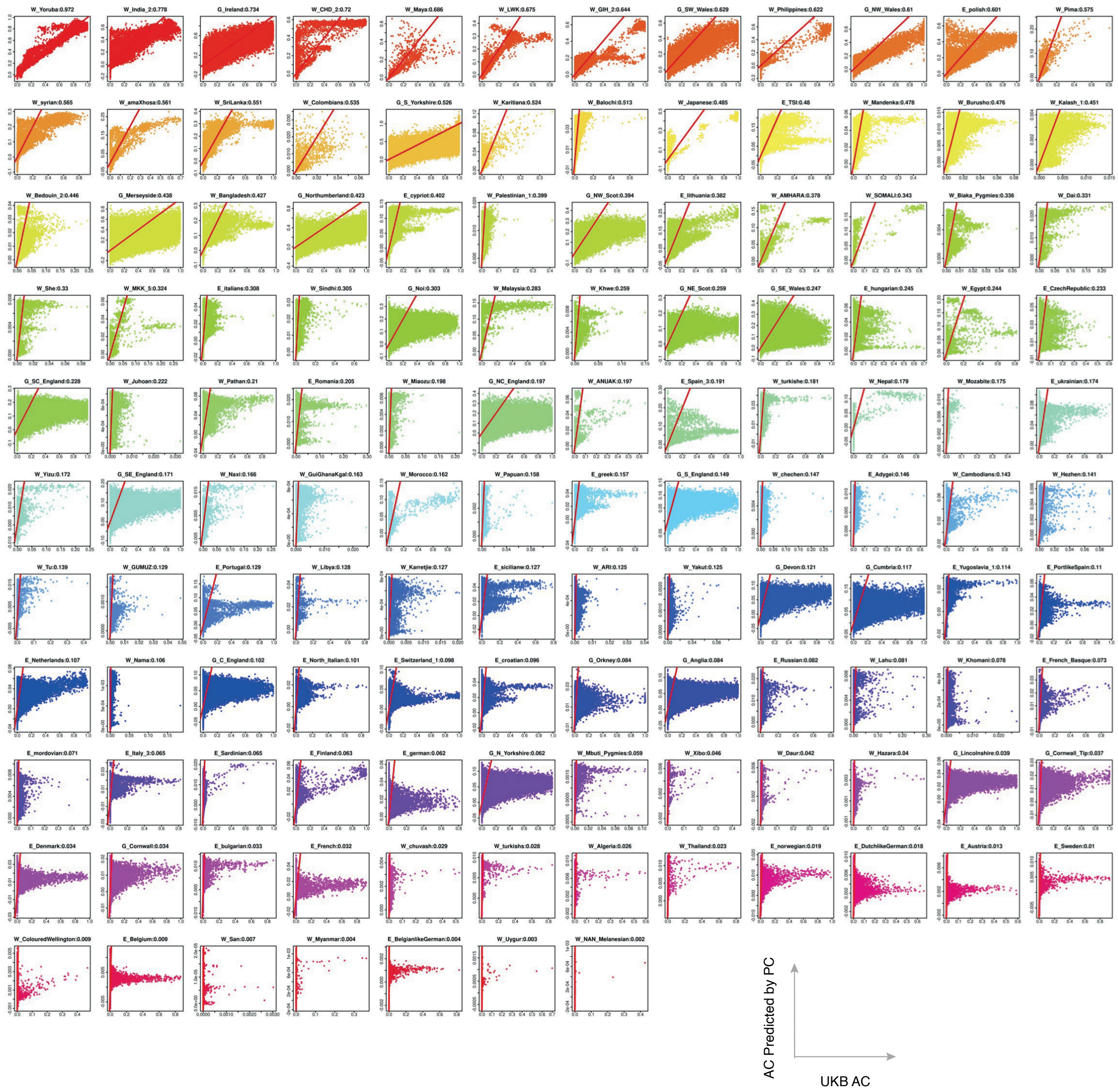

Supplementary Fig. 2. Using 140 PCs to predict 127 ACs. The X-axis shows observed ACs, and the y-axis shows PC-predicted ACs. Plots are arranged by the R^2 between the real and predicted ACs, in descending order.

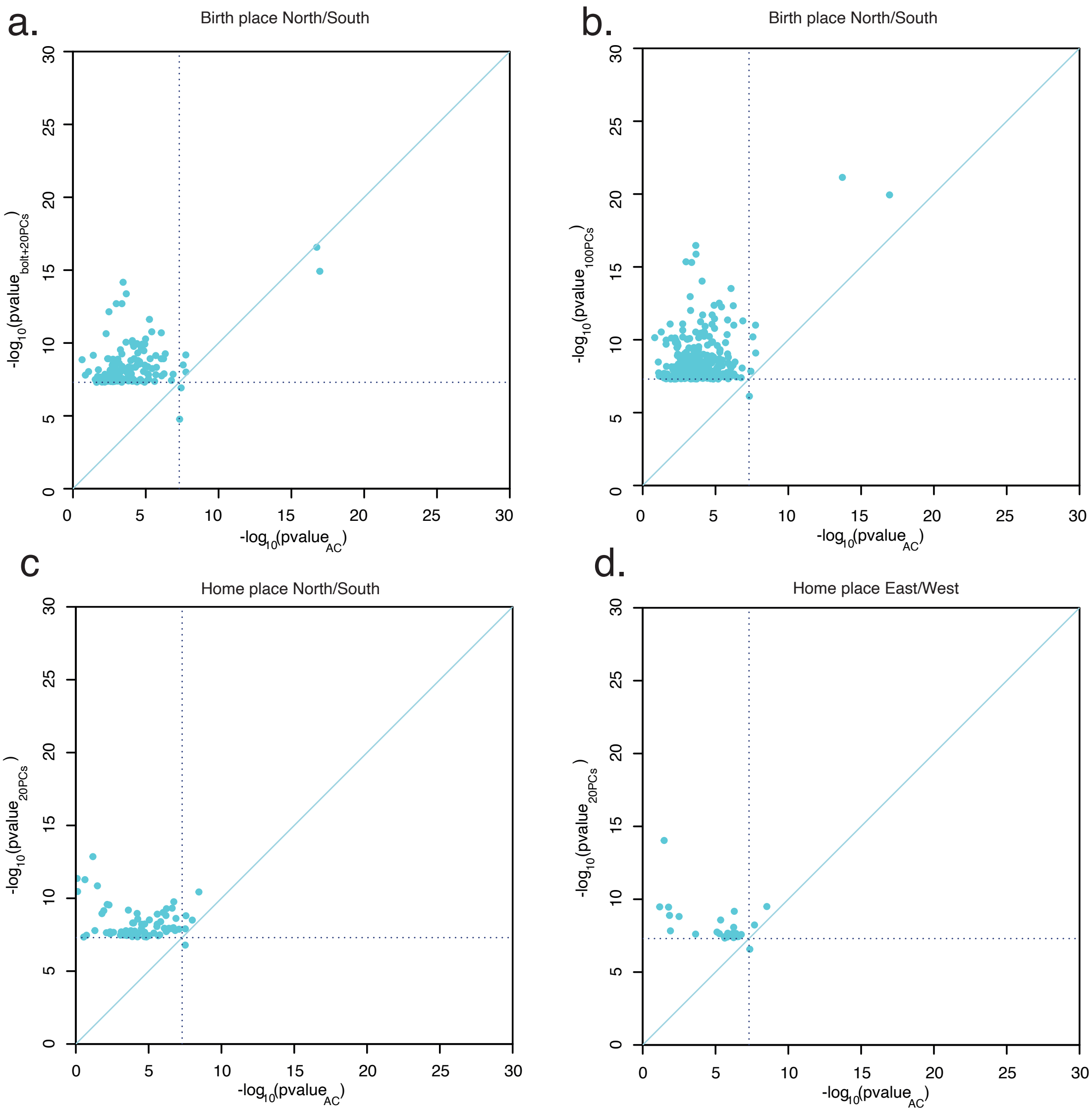

Supplementary Fig. 3. Comparison of independent GWAS hits for Place of birth (North/South) between GWAS corrected by ACs and by a) BOLT-LMM GWAS including 20 PCs as regressors; b) 100PCs, and for c) Home places (North/South) and d) Home places (East/West) between GWAS corrected by ACs and 20 PCs. Light blue coloured dots represent independent genome wide significant variants identified by each respective GWAS.

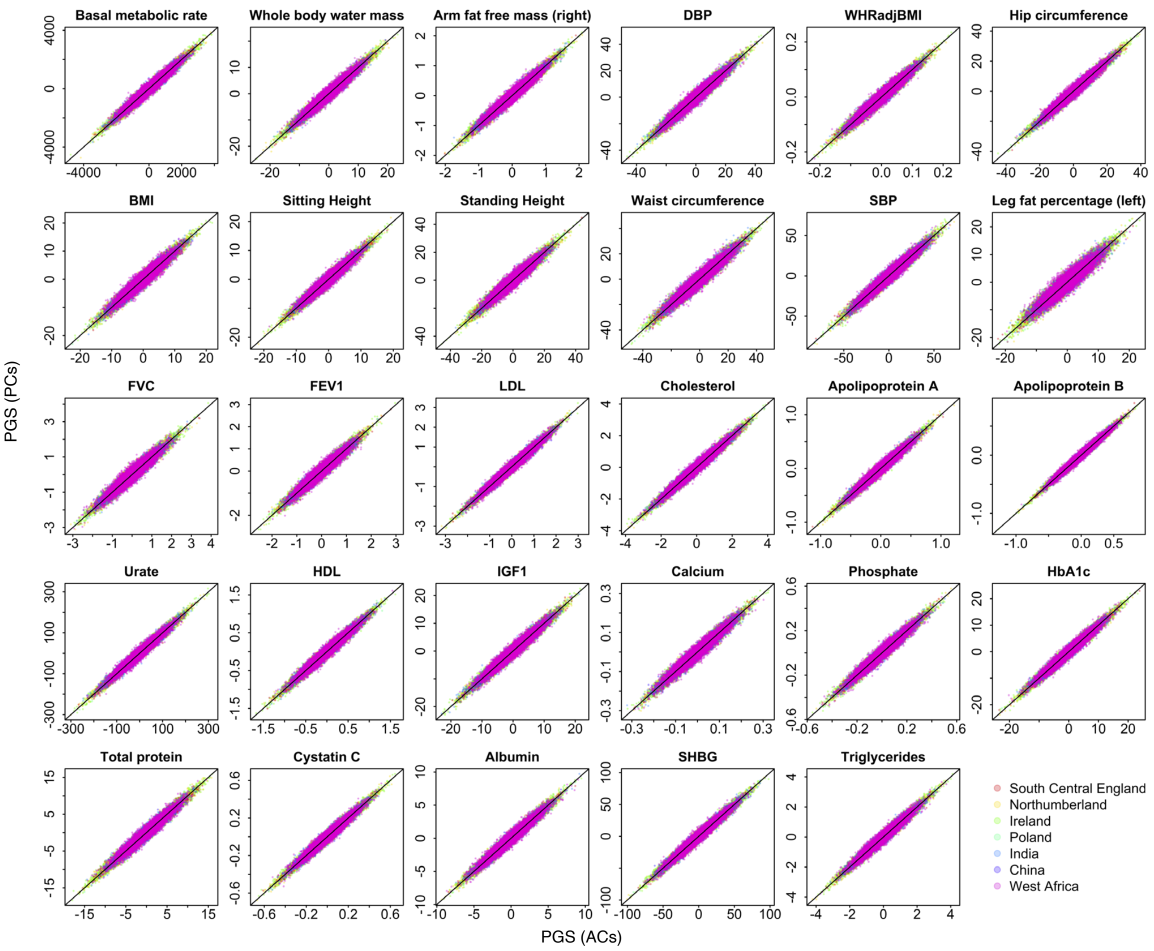

Supplementary Fig. 4. PGSs of 29 out of 53 traits constructed by both AC-corrected GWAS and PC-corrected GWAS on UKB participants, in 7 ancestral groups: South Central England, Northumberland, Ireland, Poland, India, China and West Africa. X-axes represent the PGS constructed by AC-corrected GWAS and y-axes represent the PGS constructed by PC-corrected GWAS. Each PGS was centralised by regressing out age and sex prior to plotting.

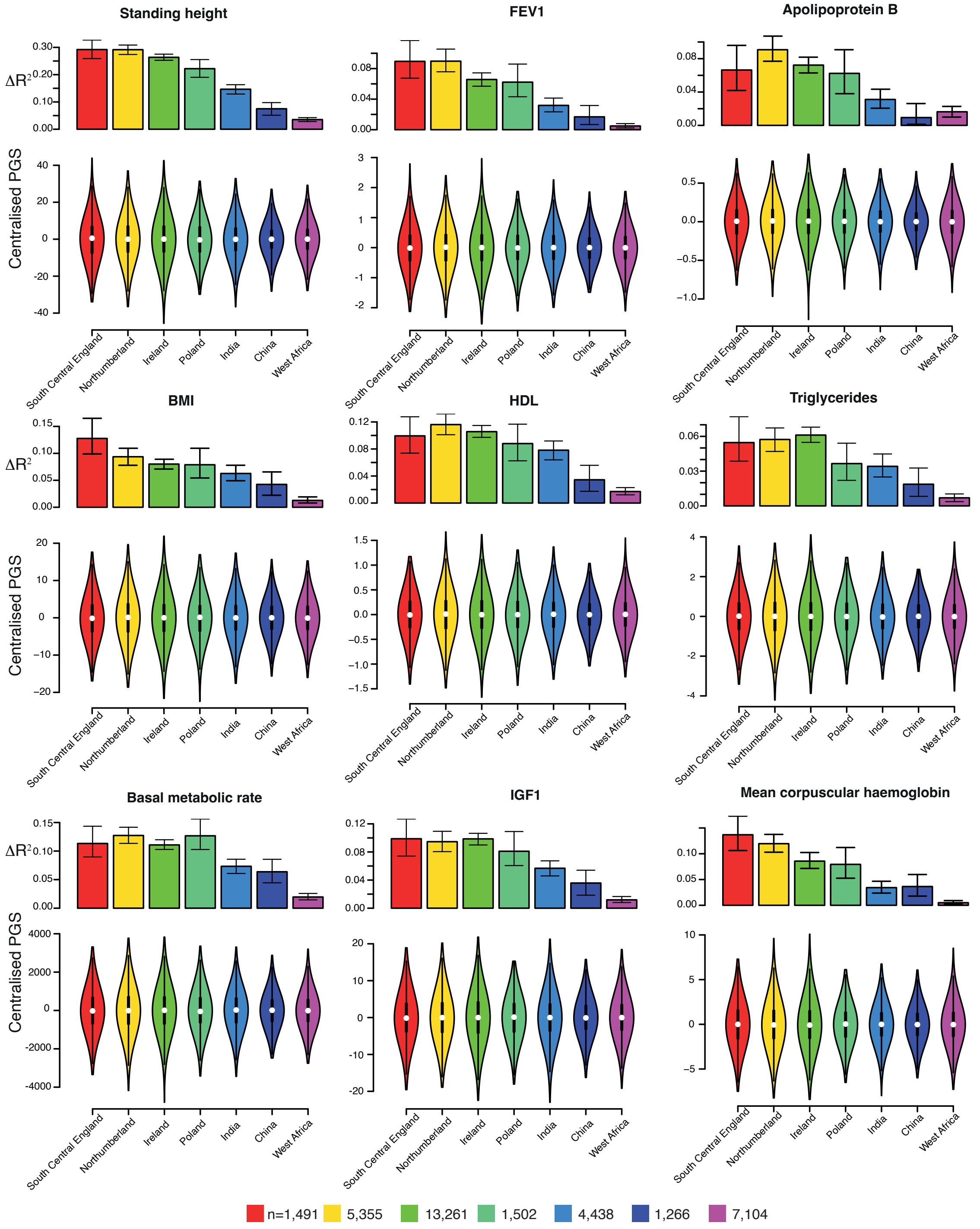

Supplementary Fig. 5. ΔR^2 (Methods) and distribution of PGSs in 7 ancestral groups across 9 out of 53 traits, using AC-corrected GWAS. Data are presented as estimated values with 95% central bootstrapped CIs. Top: ΔR^2 of PGSs in each ancestry group; bottom: the violin plots show the distribution of PGSs, across different ancestral groups. 95% confidence intervals for each bar is calculated by bootstrap using 1000 iterations.

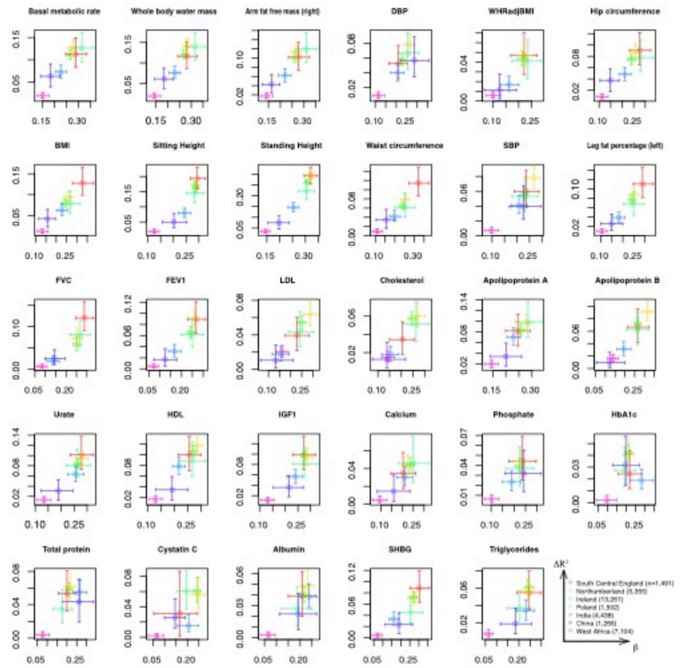

Supplementary Fig. 6. Comparison of effect size estimate of PGSs β and ΔR^2, between 7 ancestral groups across 29 out of 53 traits, using AC-corrected GWAS. Data are presented as estimated values with 95% central bootstrapped CIs. In most cases, β mirrors ΔR^2. 95% confidence intervals for each dot are calculated by bootstrap with 1000 iterations.

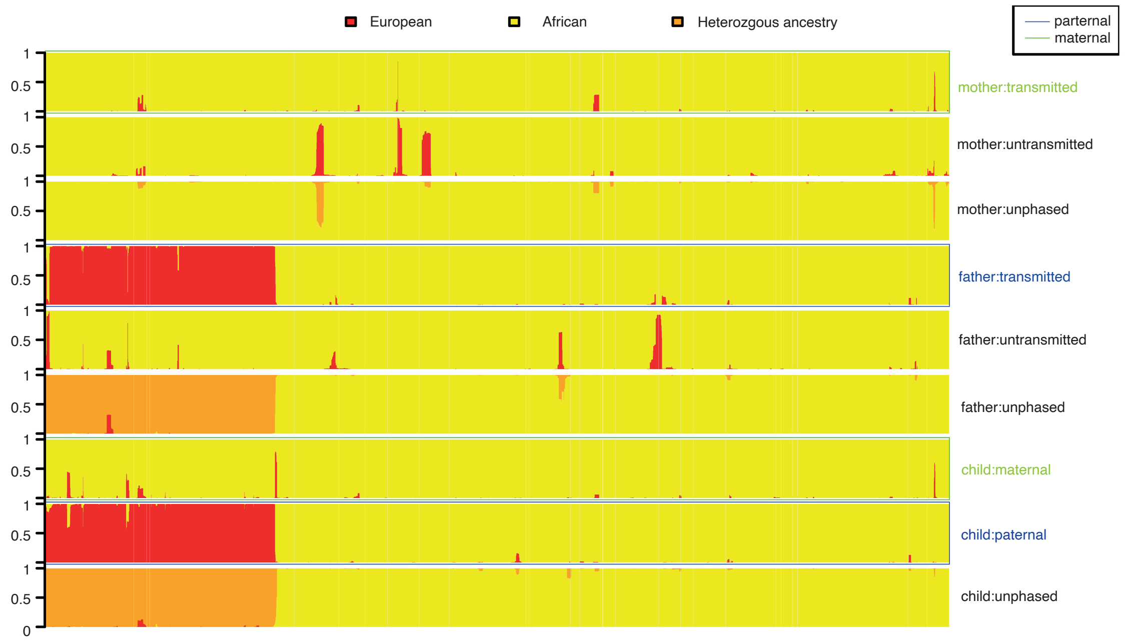

Supplementary Fig. 7. Ancestry-aware phasing of chromosome 4 in trio individuals. From bottom to top, the first three bars represent ancestry inferred using unphased genotypes, or

based on the phased paternal and then maternal haplotypes for the child in the trio; the second three bars respectively represent ancestry inferred using the unphased genotypes, un-transmitted and transmitted haplotypes for the father in the trio; the last three bars are the same, but for the mother in the trio. Ancestry inference using unphased and phased data are similar, and African (coloured in yellow), or European genome segment (red) are generally large (megabases in size).

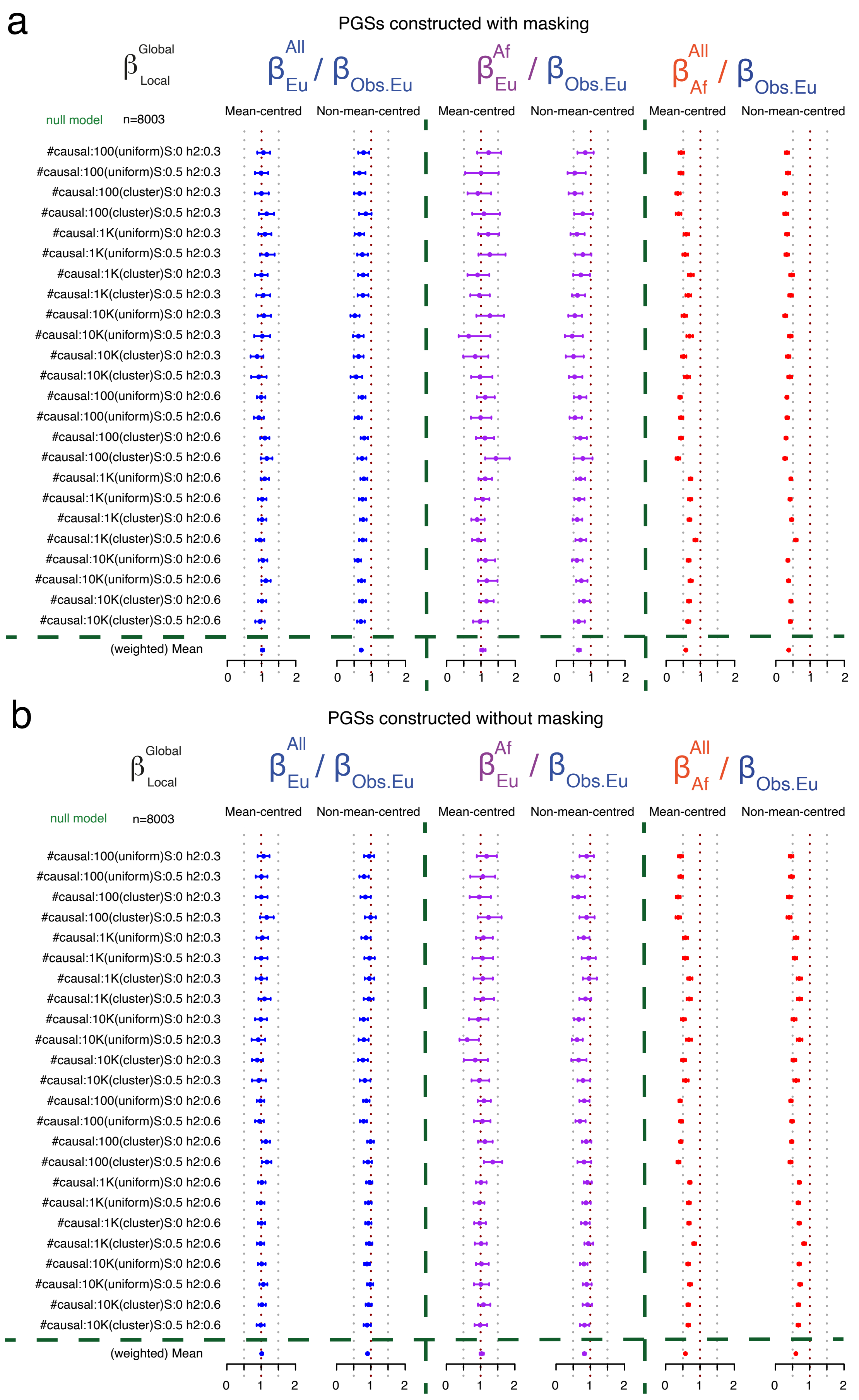
a
PGSs constructed with masking
β Global Local
β All Eu / β Obs.Eu
β Af Eu / β Obs.Eu
β All Af / β Obs.Eu
null model
n=8003
Mean-centred
Non-mean-centred
#causal:100(uniform)S:0 h2:0.3
#causal:100(uniform)S:0.5 h2:0.3
#causal:100(cluster)S:0 h2:0.3
#causal:100(cluster)S:0.5 h2:0.3
#causal:1K(uniform)S:0 h2:0.3
#causal:1K(uniform)S:0.5 h2:0.3
#causal:1K(cluster)S:0 h2:0.3
#causal:1K(cluster)S:0.5 h2:0.3
#causal:10K(uniform)S:0 h2:0.3
#causal:10K(uniform)S:0.5 h2:0.3
#causal:10K(cluster)S:0 h2:0.3
#causal:10K(cluster)S:0.5 h2:0.3
#causal:100(uniform)S:0 h2:0.6
#causal:100(uniform)S:0.5 h2:0.6
#causal:100(cluster)S:0 h2:0.6
#causal:100(cluster)S:0.5 h2:0.6
#causal:1K(uniform)S:0 h2:0.6
#causal:1K(uniform)S:0.5 h2:0.6
#causal:1K(cluster)S:0 h2:0.6
#causal:1K(cluster)S:0.5 h2:0.6
#causal:10K(uniform)S:0 h2:0.6
#causal:10K(uniform)S:0.5 h2:0.6
#causal:10K(cluster)S:0 h2:0.6
#causal:10K(cluster)S:0.5 h2:0.6
(weighted) Mean
0 1 2
b
PGSs constructed without masking
β Global Local
β All Eu / β Obs.Eu
β Af Eu / β Obs.Eu
β All Af / β Obs.Eu
null model
n=8003
Mean-centred
Non-mean-centred
#causal:100(uniform)S:0 h2:0.3
#causal:100(uniform)S:0.5 h2:0.3
#causal:100(cluster)S:0 h2:0.3
#causal:100(cluster)S:0.5 h2:0.3
#causal:1K(uniform)S:0 h2:0.3
#causal:1K(uniform)S:0.5 h2:0.3
#causal:1K(cluster)S:0 h2:0.3
#causal:1K(cluster)S:0.5 h2:0.3
#causal:10K(uniform)S:0 h2:0.3
#causal:10K(uniform)S:0.5 h2:0.3
#causal:10K(cluster)S:0 h2:0.3
#causal:10K(cluster)S:0.5 h2:0.3
#causal:100(uniform)S:0 h2:0.6
#causal:100(uniform)S:0.5 h2:0.6
#causal:100(cluster)S:0 h2:0.6
#causal:100(cluster)S:0.5 h2:0.6
#causal:1K(uniform)S:0 h2:0.6
#causal:1K(uniform)S:0.5 h2:0.6
#causal:1K(cluster)S:0 h2:0.6
#causal:1K(cluster)S:0.5 h2:0.6
#causal:10K(uniform)S:0 h2:0.6
#causal:10K(uniform)S:0.5 h2:0.6
#causal:10K(cluster)S:0 h2:0.6
#causal:10K(cluster)S:0.5 h2:0.6
(weighted) Mean
0 1 2

Supplementary Fig. 8. ANCHOR results for each simulated trait under the null model simulation. Data are presented as estimated values with 95% central bootstrapped CIs. a) PGSs are constructed with masking uncertain and short segments and b). PGSs are constructed without masking uncertain and short segments. Every two columns separated by the vertical green dash line show ratios of estimates defined as in Main Figure 5. Odd columns show result with PGSs constructed by "mean-centring" and even columns show result with PGSs constructed without "mean-centring". Rows show simulated phenotypes. The final row shows combined estimates. The first and second two columns estimate the underlying correlation in causal effect sizes between European-ancestry and either all 8003 African-ancestry individuals, or individuals of 100% African-ancestry, respectively, while the third two columns additionally estimate the impact of local effects on predictive power for African-ancestry segments.

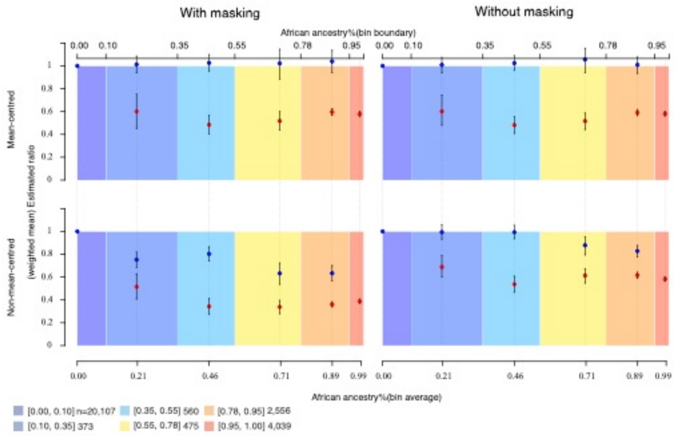

Supplementary Fig. 9. Results of application of ANCHOR for 24 UKB simulated traits, for individuals of varying African ancestry binned as in main Figure 4c (x-axis; coloured region). Data are presented as (weighted) means with 95% central bootstrapped CIs. For each bin in each panel, estimates of coefficients $\beta_{Eu}/\beta_{Obs.Eu}$ (blue) and $\beta_{Af}/\beta_{Obs.Eu}$ (red) are shown with 95% bootstrapped confidence intervals, representing the ratio of PGS within European and African genomic regions to the PGS obtained from external European samples respectively (Methods). Also shown are these estimates from individuals of ~100% European ($\beta_{Obs.Eu}$; blue horizontal bar) or ~100% African (red horizontal bar) ancestry. Each panel represent one of the combinations of with/without mean-centring and with/without masking the uncertain and short segments for 24 simulated traits under the null model simulation: panels in the top represent PGS constructed with mean centring, and panels in the bottom represent PGS

constructed without mean-centring; panels in the left represent PGS constructed with masking the uncertain and short segments and panels in the right represent PGS constructed without masking the uncertain and short segments.

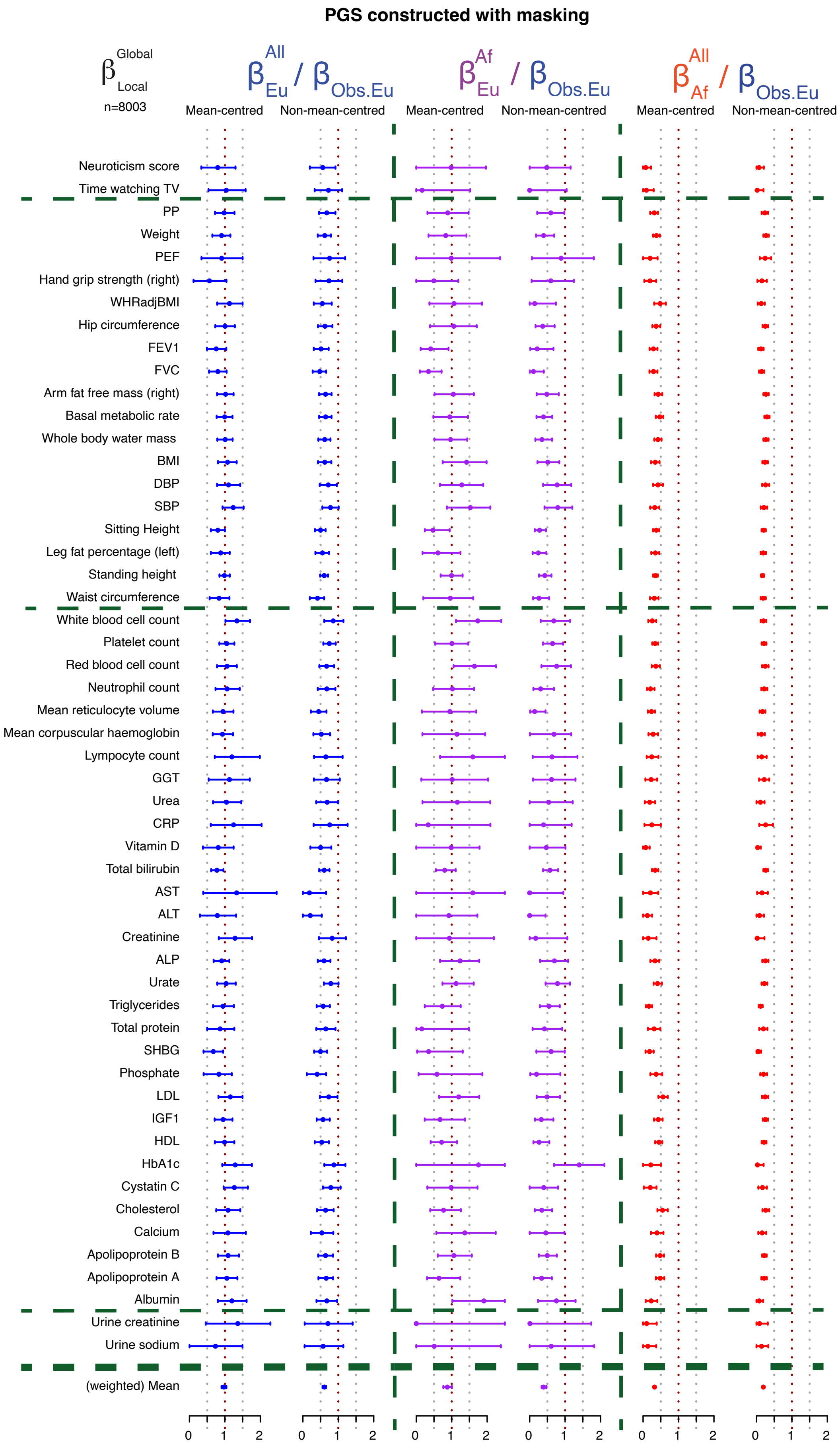

PGS constructed with masking
β Global / Local
n=8003
β All Eu / β Obs.Eu
β Af Eu / β Obs.Eu
β All Af / β Obs.Eu
Mean-centred
Non-mean-centred
Neuroticism score
Time watching TV
PP
Weight
PEF
Hand grip strength (right)
WHRadjBMI
Hip circumference
FEV1
FVC
Arm fat free mass (right)
Basal metabolic rate
Whole body water mass
BMI
DBP
SBP
Sitting Height
Leg fat percentage (left)
Standing height
Waist circumference
White blood cell count
Platelet count
Red blood cell count
Neutrophil count
Mean reticulocyte volume
Mean corpuscular haemoglobin
Lympocyte count
GGT
Urea
CRP
Vitamin D
Total bilirubin
AST
ALT
Creatinine
ALP
Urate
Triglycerides
Total protein
SHBG
Phosphate
LDL
IGF1
HDL
HbA1c
Cystatin C
Cholesterol
Calcium
Apolipoprotein B
Apolipoprotein A
Albumin
Urine creatinine
Urine sodium
(weighted) Mean
0 1 2

Supplementary Fig. 10. ANCHOR results for each UKB traits. Data are presented as estimated values with 95% central bootstrapped CIs. PGSs are constructed with masking uncertain and short segments. Every two columns separated by the vertical green dash line show ratios of estimates defined as in Main Figure 5. Odd columns show result with PGSs constructed by "mean-centring" and even columns show result with PGSs constructed without "mean-centring". Rows show simulated phenotypes. The final row shows combined estimates. The first and second two columns estimate the underlying correlation in causal effect sizes between European-ancestry and either all 8003 African-ancestry individuals, or individuals of 100% African-ancestry, respectively, while the third two columns additionally estimate the impact of local effects on predictive power for African-ancestry segments.

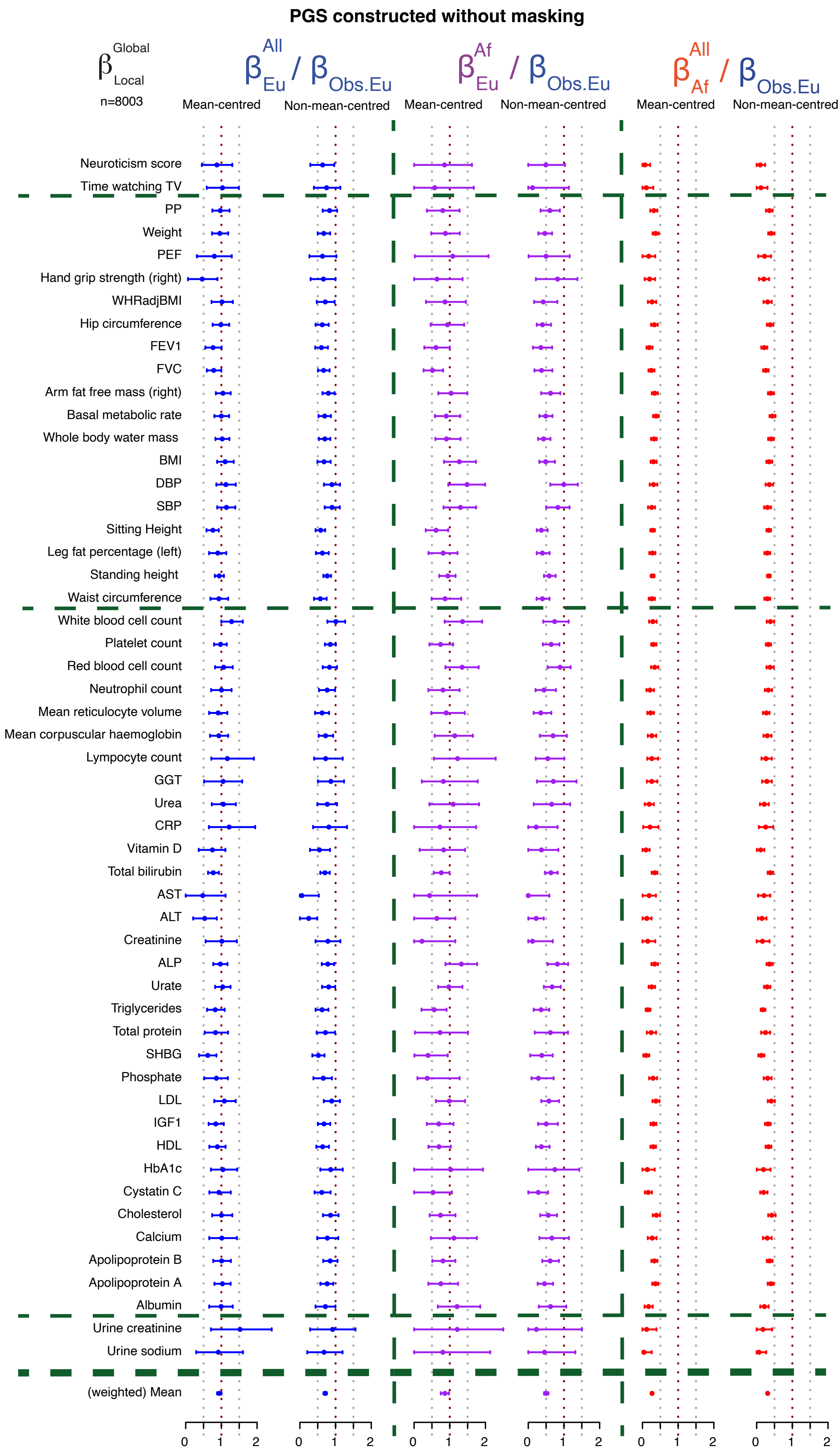

PGS constructed without masking
β Global / Local
n=8003
β All Eu / β Obs.Eu
β Af Eu / β Obs.Eu
β All Af / β Obs.Eu
Mean-centred
Non-mean-centred
Neuroticism score
Time watching TV
PP
Weight
PEF
Hand grip strength (right)
WHRadjBMI
Hip circumference
FEV1
FVC
Arm fat free mass (right)
Basal metabolic rate
Whole body water mass
BMI
DBP
SBP
Sitting Height
Leg fat percentage (left)
Standing height
Waist circumference
White blood cell count
Platelet count
Red blood cell count
Neutrophil count
Mean reticulocyte volume
Mean corpuscular haemoglobin
Lympocyte count
GGT
Urea
CRP
Vitamin D
Total bilirubin
AST
ALT
Creatinine
ALP
Urate
Triglycerides
Total protein
SHBG
Phosphate
LDL
IGF1
HDL
HbA1c
Cystatin C
Cholesterol
Calcium
Apolipoprotein B
Apolipoprotein A
Albumin
Urine creatinine
Urine sodium
(weighted) Mean
0
1
2

Supplementary Fig. 11. ANCHOR results for each UKB traits. PGSs are constructed without masking uncertain and short segments. Data are presented as estimated values with 95% central bootstrapped CIs. Every two columns separated by the vertical green dash line show ratios of estimates defined as in Main Figure 5. Odd columns show result with PGSs constructed by "mean-centring" and even columns show result with PGSs constructed without "mean-centring". Rows show simulated phenotypes. The final row shows combined estimates. The first and second two columns estimate the underlying correlation in causal effect sizes between European-ancestry and either all 8003 African-ancestry individuals, or individuals of 100% African-ancestry, respectively, while the third two columns additionally estimate the impact of local effects on predictive power for African-ancestry segments.

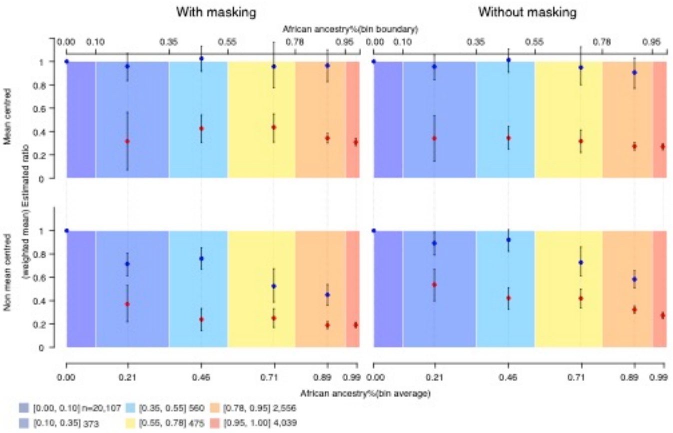

Supplementary Fig. 12. Results of application of ANCHOR for 53 UKB traits, for individuals of varying African ancestry binned as in main Figure 4c (x-axis; coloured region). Data are presented as (weighted) means with 95% central bootstrapped CIs. For each bin in each panel, estimates of coefficients $\beta_{Eu}/\beta_{Obs.Eu}$ (blue) and $\beta_{Af}/\beta_{Obs.Eu}$ (red) are shown with 95% bootstrapped confidence intervals, representing the ratio of PGS within European and African genomic regions to the PGS obtained from external European samples respectively (Methods). Also shown are these estimates from individuals of ~100% European ($\beta_{Obs.Eu}$; blue horizontal bar) or ~100% African (red horizontal bar) ancestry. Each panel represent one of the combinations of with/without mean-centring and with/without masking the uncertain and short segments for 53 UKB traits: panels in the top represent PGS constructed with mean centring, and panels in the bottom represent PGS constructed without mean-centring; panels in the left represent PGS constructed with masking the uncertain and short segments and

panels in the right represent PGS constructed without masking the uncertain and short segments.

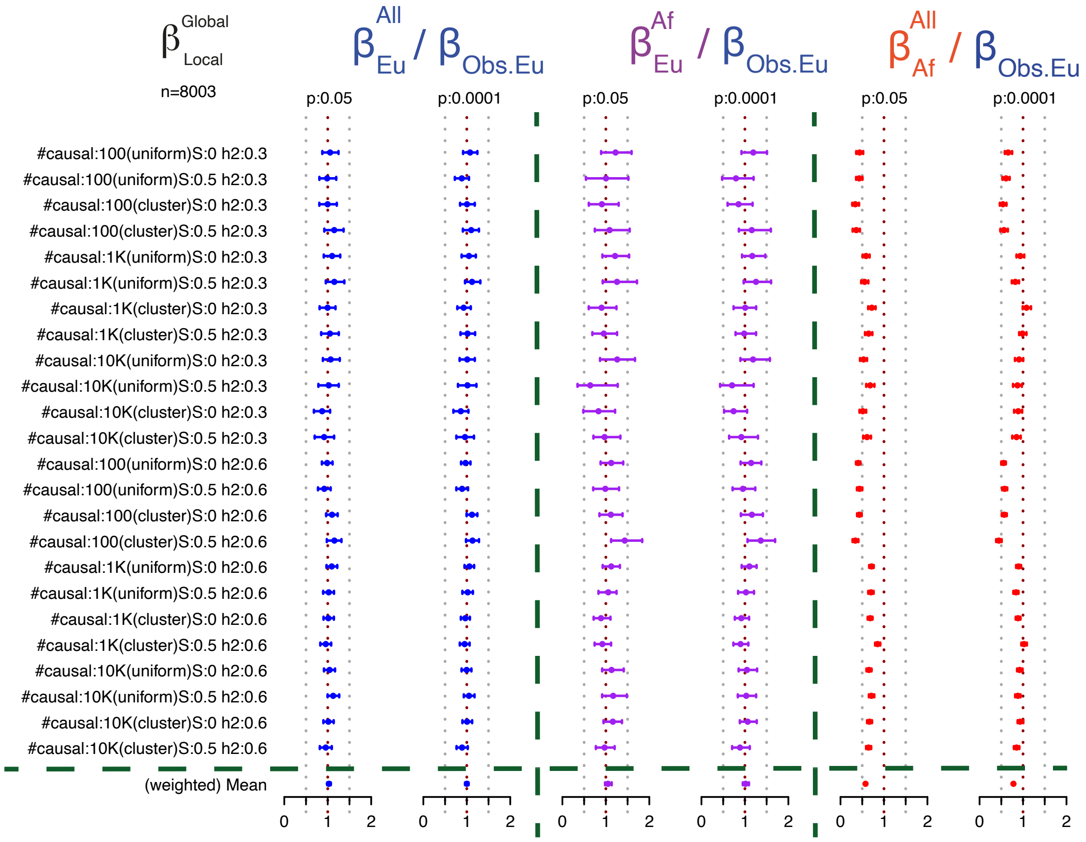

Supplementary Fig. 13. ANCHOR results for all the 24 simulated traits under null model. Data are presented as estimated values with 95% central bootstrapped CIs. Every two columns separated by the vertical green dash line show ratios of estimates defined as in Main Figure 5. Odd columns show result with PGSs constructed by using default p-value 0.05 in thresholding and even columns show result with PGSs constructed by using alternative p-value 0.0001 in thresholding. Rows show each of 24 simulated phenotypes. The final two row show combined estimates. The first and second two columns estimate the underlying correlation in causal effect sizes between European-ancestry and either all 8003 African-ancestry individuals, or individuals of 100% African-ancestry, respectively, while the third two columns additionally estimate the impact of local effects on predictive power for African-ancestry segments. Overall the ratio estimation from two different p-value thresholds in PGS construction are very consistent across most of the traits.

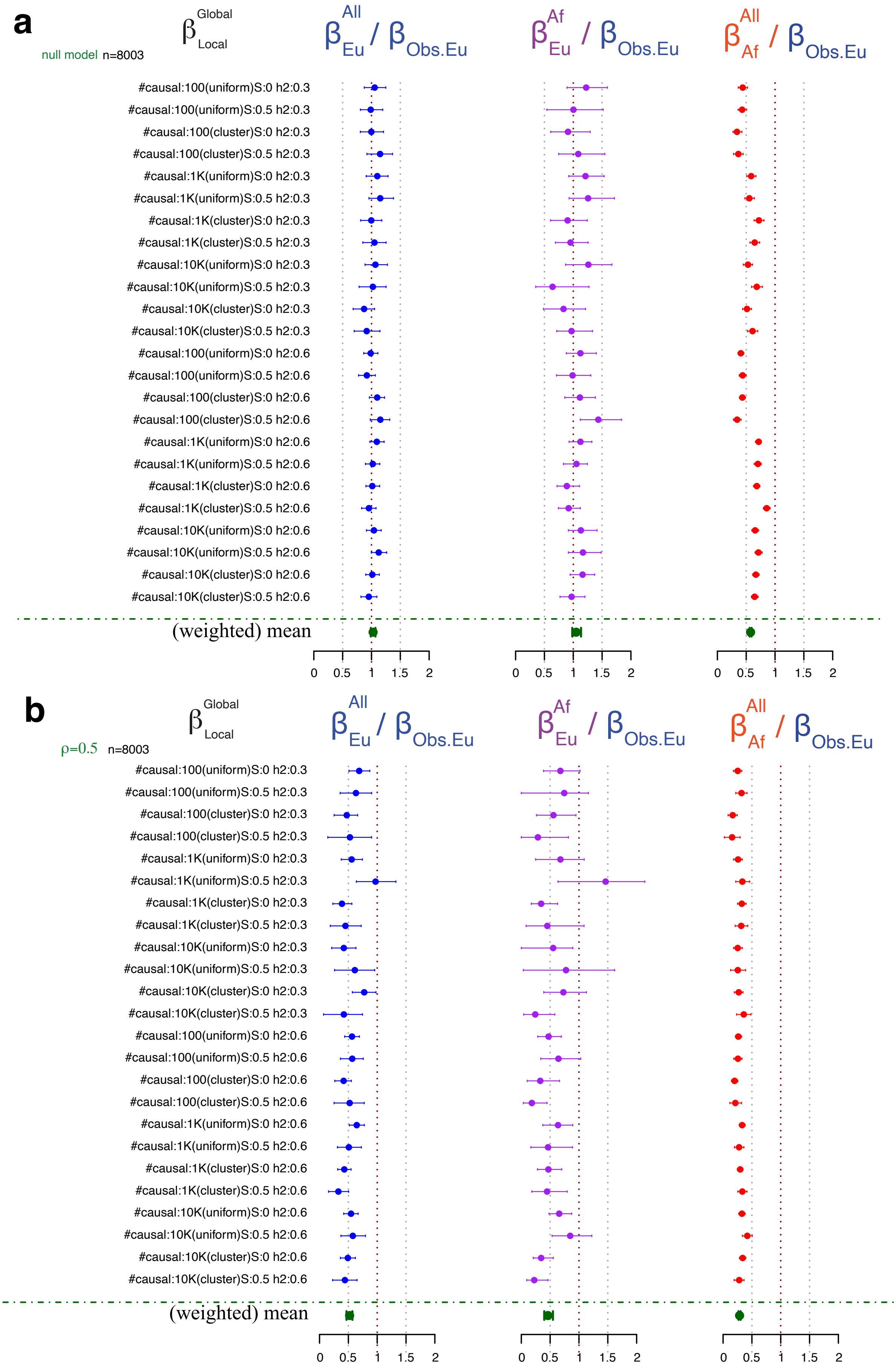
a
null model n=8003
β Global Local
β All Eu / β Obs.Eu
β Af Eu / β Obs.Eu
β All Af / β Obs.Eu
#causal:100(uniform)S:0 h2:0.3
#causal:100(uniform)S:0.5 h2:0.3
#causal:100(cluster)S:0 h2:0.3
#causal:100(cluster)S:0.5 h2:0.3
#causal:1K(uniform)S:0 h2:0.3
#causal:1K(uniform)S:0.5 h2:0.3
#causal:1K(cluster)S:0 h2:0.3
#causal:1K(cluster)S:0.5 h2:0.3
#causal:10K(uniform)S:0 h2:0.3
#causal:10K(uniform)S:0.5 h2:0.3
#causal:10K(cluster)S:0 h2:0.3
#causal:10K(cluster)S:0.5 h2:0.3
#causal:100(uniform)S:0 h2:0.6
#causal:100(uniform)S:0.5 h2:0.6
#causal:100(cluster)S:0 h2:0.6
#causal:100(cluster)S:0.5 h2:0.6
#causal:1K(uniform)S:0 h2:0.6
#causal:1K(uniform)S:0.5 h2:0.6
#causal:1K(cluster)S:0 h2:0.6
#causal:1K(cluster)S:0.5 h2:0.6
#causal:10K(uniform)S:0 h2:0.6
#causal:10K(uniform)S:0.5 h2:0.6
#causal:10K(cluster)S:0 h2:0.6
#causal:10K(cluster)S:0.5 h2:0.6
(weighted) mean
0 0.5 1 1.5 2
b
ρ=0.5 n=8003
β Global Local
β All Eu / β Obs.Eu
β Af Eu / β Obs.Eu
β All Af / β Obs.Eu
#causal:100(uniform)S:0 h2:0.3
#causal:100(uniform)S:0.5 h2:0.3
#causal:100(cluster)S:0 h2:0.3
#causal:100(cluster)S:0.5 h2:0.3
#causal:1K(uniform)S:0 h2:0.3
#causal:1K(uniform)S:0.5 h2:0.3
#causal:1K(cluster)S:0 h2:0.3
#causal:1K(cluster)S:0.5 h2:0.3
#causal:10K(uniform)S:0 h2:0.3
#causal:10K(uniform)S:0.5 h2:0.3
#causal:10K(cluster)S:0 h2:0.3
#causal:10K(cluster)S:0.5 h2:0.3
#causal:100(uniform)S:0 h2:0.6
#causal:100(uniform)S:0.5 h2:0.6
#causal:100(cluster)S:0 h2:0.6
#causal:100(cluster)S:0.5 h2:0.6
#causal:1K(uniform)S:0 h2:0.6
#causal:1K(uniform)S:0.5 h2:0.6
#causal:1K(cluster)S:0 h2:0.6
#causal:1K(cluster)S:0.5 h2:0.6
#causal:10K(uniform)S:0 h2:0.6
#causal:10K(uniform)S:0.5 h2:0.6
#causal:10K(cluster)S:0 h2:0.6
#causal:10K(cluster)S:0.5 h2:0.6
(weighted) mean
0 0.5 1 1.5 2

Supplementary Fig. 14. ANCHOR results for each simulated trait. Columns show ratios of estimates defined as in Main Figure 5, and rows show simulated phenotypes. Data are presented as estimated values with 95% central bootstrapped CIs. The final row shows combined estimates. The first and second columns estimate the underlying correlation in causal effect sizes between European-ancestry and either all 8003 African-ancestry individuals, or individuals of 100% African-ancestry, respectively, while the third column additionally estimate the impact of local effects on predictive power for African-ancestry segments in the same two settings. a) Results under the null model $\rho=1$, and b) results under the model $\rho=0.5$, with incomplete correlation in underlying effects sizes between different populations.

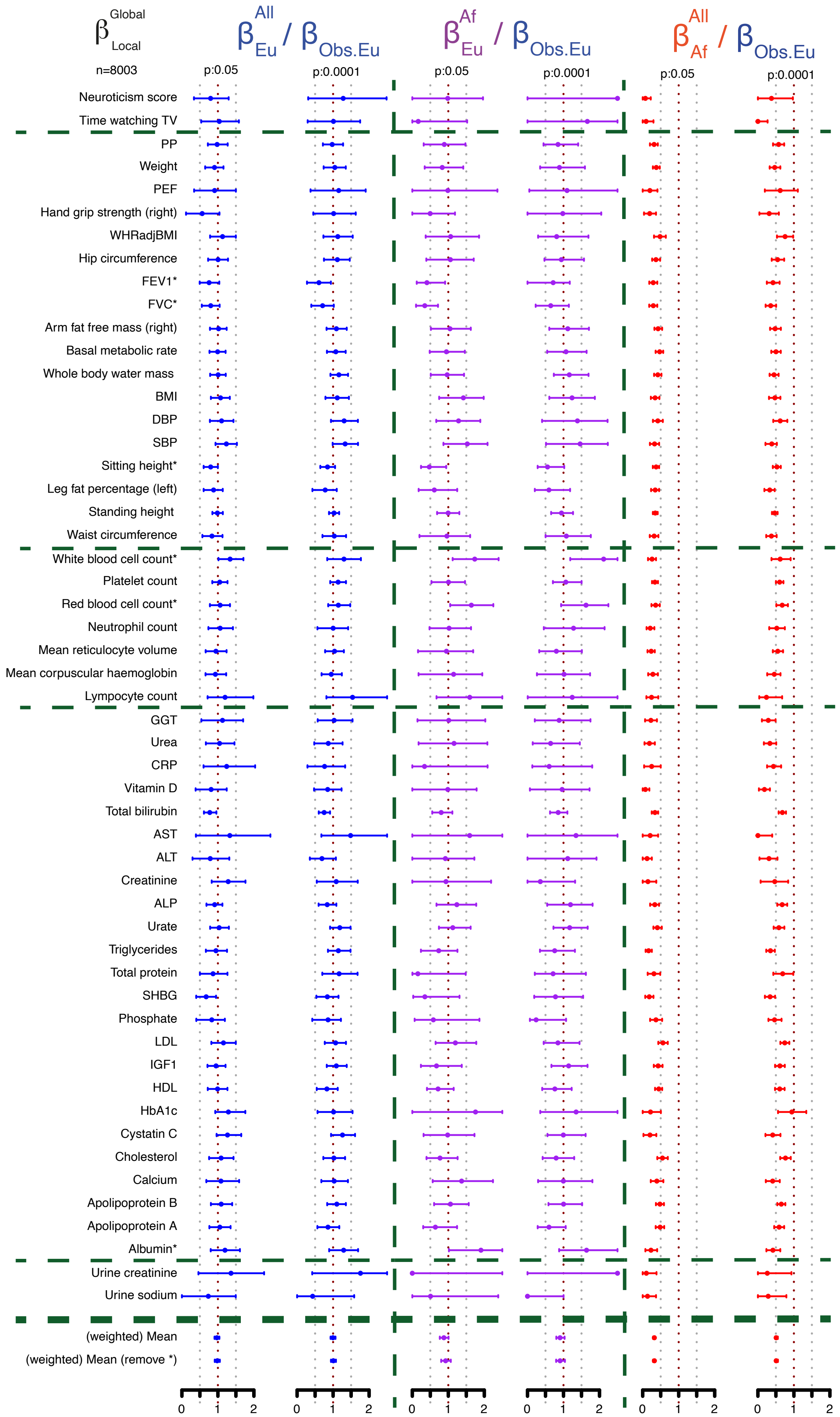

β Global Local
n=8003
β All Eu / β Obs.Eu
β Af Eu / β Obs.Eu
β All Af / β Obs.Eu
p:0.05
p:0.0001
Neuroticism score
Time watching TV
PP
Weight
PEF
Hand grip strength (right)
WHRadjBMI
Hip circumference
FEV1*
FVC*
Arm fat free mass (right)
Basal metabolic rate
Whole body water mass
BMI
DBP
SBP
Sitting height*
Leg fat percentage (left)
Standing height
Waist circumference
White blood cell count*
Platelet count
Red blood cell count*
Neutrophil count
Mean reticulocyte volume
Mean corpuscular haemoglobin
Lympocyte count
GGT
Urea
CRP
Vitamin D
Total bilirubin
AST
ALT
Creatinine
ALP
Urate
Triglycerides
Total protein
SHBG
Phosphate
LDL
IGF1
HDL
HbA1c
Cystatin C
Cholesterol
Calcium
Apolipoprotein B
Apolipoprotein A
Albumin*
Urine creatinine
Urine sodium
(weighted) Mean
(weighted) Mean (remove *)
0 1 2

Supplementary Fig. 15. ANCHOR results for all the 53 UKB traits. Every two columns separated by the vertical green dash line show ratios of estimates defined as in Main Figure 5. Data are presented as estimated values with 95% central bootstrapped CIs. Odd columns show result with PGSs constructed by using default p-value 0.05 in thresholding and even columns show result with PGSs constructed by using alternative p-value 0.0001 in thresholding. Rows show each of 53 UKB phenotypes. The final two row show combined estimates. The first and second two columns estimate the underlying correlation in causal effect sizes between European-ancestry and either all 8003 African-ancestry individuals, or individuals of 100% African-ancestry, respectively, while the third two columns additionally estimate the impact of local effects on predictive power for African-ancestry segments. Overall the ratio estimation from two different p-value thresholds in PGS construction are very consistent across most of the traits.

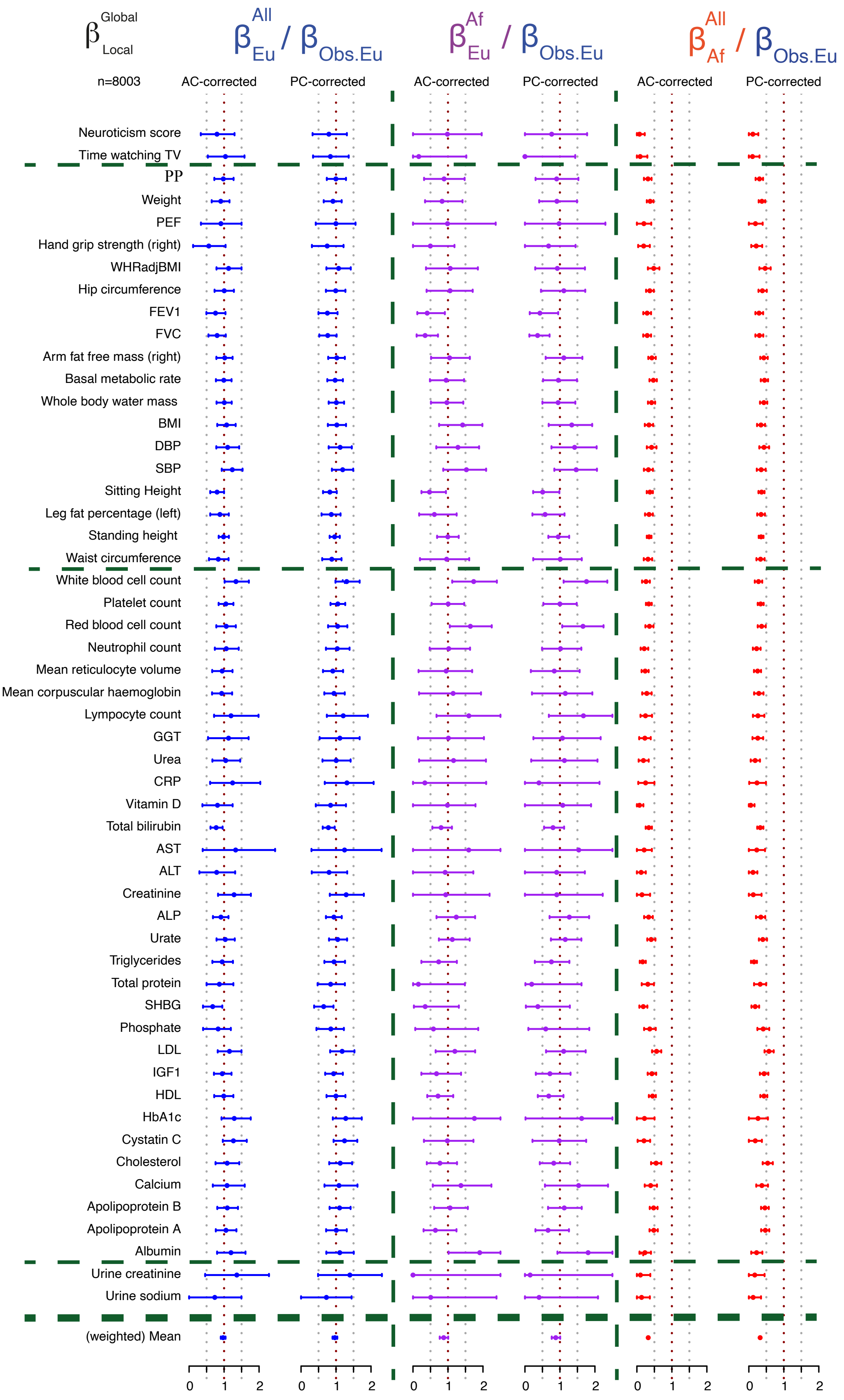

β Global Local
n=8003
β All Eu / β Obs.Eu
β Af Eu / β Obs.Eu
β All Af / β Obs.Eu
AC-corrected
PC-corrected
Neuroticism score
Time watching TV
PP
Weight
PEF
Hand grip strength (right)
WHRadjBMI
Hip circumference
FEV1
FVC
Arm fat free mass (right)
Basal metabolic rate
Whole body water mass
BMI
DBP
SBP
Sitting Height
Leg fat percentage (left)
Standing height
Waist circumference
White blood cell count
Platelet count
Red blood cell count
Neutrophil count
Mean reticulocyte volume
Mean corpuscular haemoglobin
Lympocyte count
GGT
Urea
CRP
Vitamin D
Total bilirubin
AST
ALT
Creatinine
ALP
Urate
Triglycerides
Total protein
SHBG
Phosphate
LDL
IGF1
HDL
HbA1c
Cystatin C
Cholesterol
Calcium
Apolipoprotein B
Apolipoprotein A
Albumin
Urine creatinine
Urine sodium
(weighted) Mean
0 1 2

Supplementary Fig. 16. ANCHOR results for all the 53 UKB traits. Every two columns separated by the vertical green dash line show ratios of estimates defined as in Main Figure 5. Data are presented as estimated values with 95% central bootstrapped CIs. Odd columns show result with PGSs constructed by using AC-corrected GWAS and even columns show result with PGSs constructed by using PC-corrected GWAS. Rows show each of 53 UKB phenotypes. The final two row show combined estimates. The first and second two columns estimate the underlying correlation in causal effect sizes between European-ancestry and either all 8003 African-ancestry individuals, or individuals of 100% African-ancestry, respectively, while the third two columns additionally estimate the impact of local effects on predictive power for African-ancestry segments. Overall the ratio estimation from two different methods of correcting population stratification in PGS construction are very consistent across most of the traits.

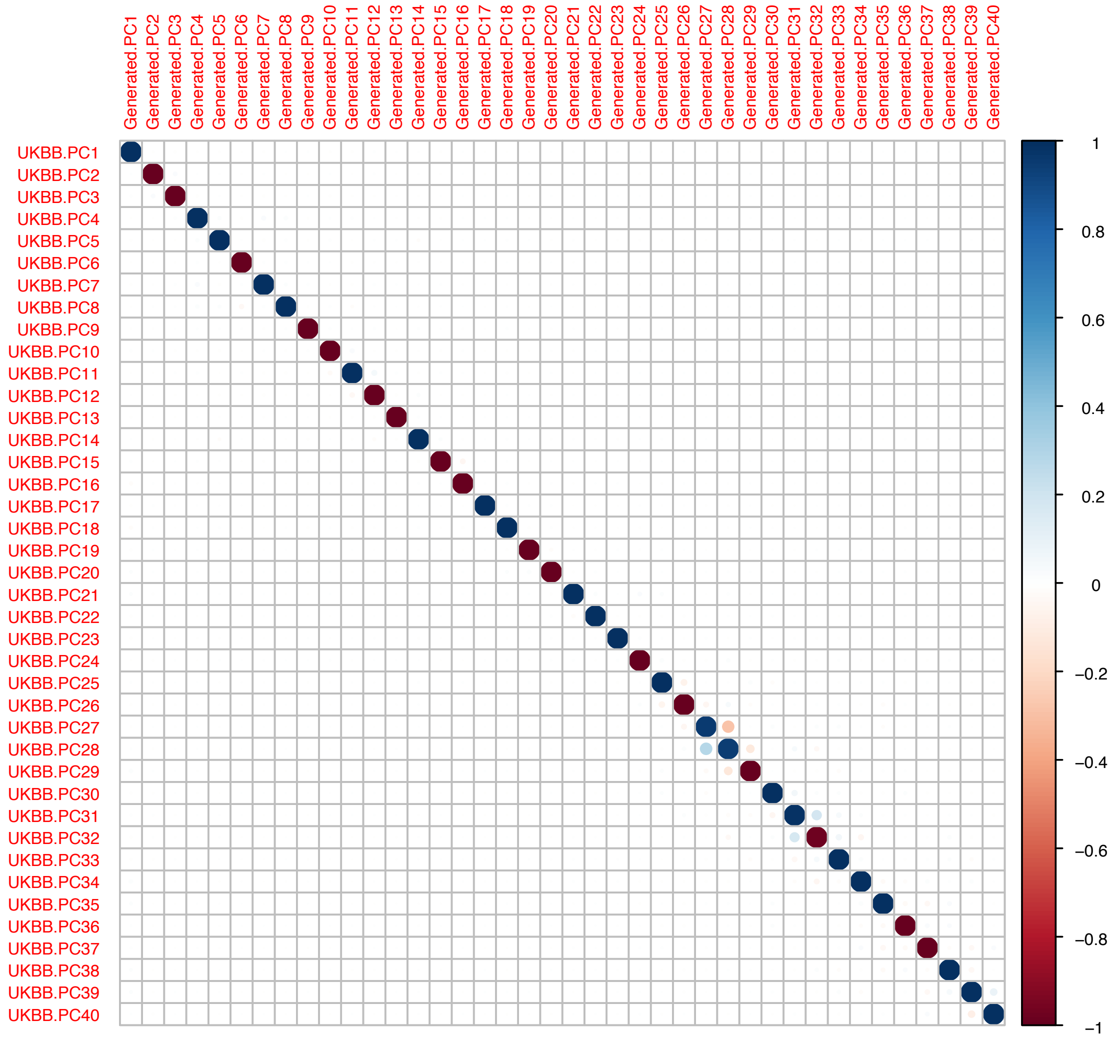

Supplementary Fig. 17. Comparison of 40PCs released by UKB, and 40PCs generated as described in Methods, to verify very high correlations (either close to 1, or close to minus 1; the sign of each PC is arbitrary).

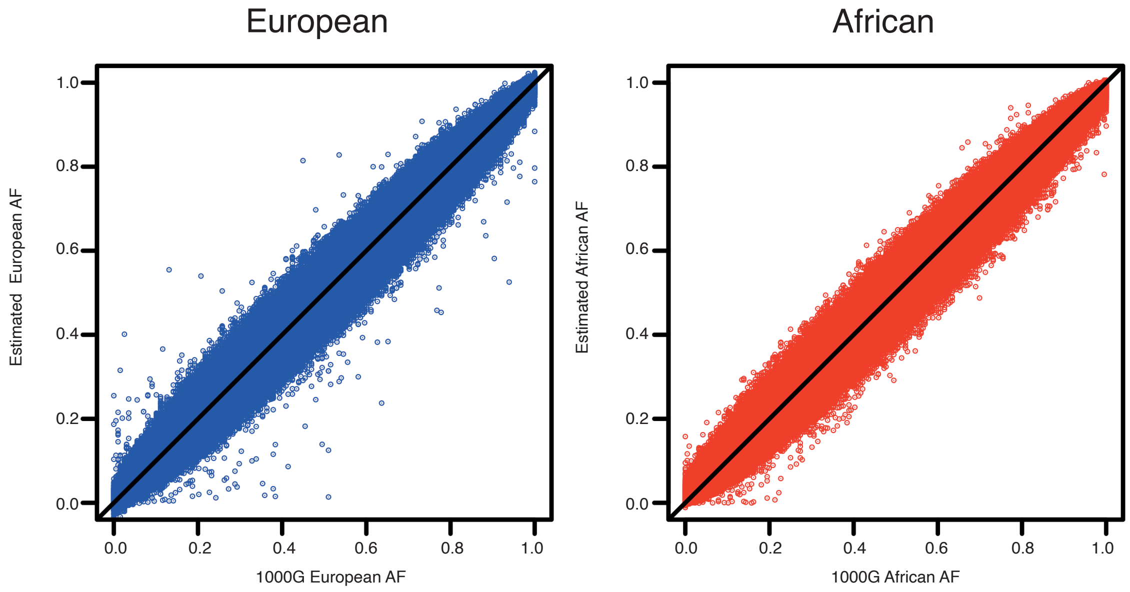

Supplementary Fig. 18. Comparison of effect allele frequency in 1000G cohort and effect allele frequency estimated by ANCHOR. Left: Effect allele frequency in European (blue dots); right: Effect allele frequency in African (red dots).

Supplementary note 1

Table of Contents

Ancestry pipeline *2*
Data Preparation 2
References 6
Phenotypes in the UKB *8*
Definition of EA 8
Definition of SBP, DBP and PP 8
Definition of eGFR 9
References 9
Genetic ancestry in the UKB *10*
Visualisation of the inferred genetic ancestry/entropy of UK-born UKB samples 10
Summary of entropy of UKB participants born in the UK/Ireland 10
Reference 11
Estimation of regional allele frequencies in the UK and worldwide *12*
Expectation-maximization based method to estimate regional allele frequency 12
Variant rs5743618 13
GWAS analysis *14*
Running BOLT-LMM for place of birth (North/South) 14
GWAS with different number of PCs 14
References 16
Portability of polygenic score across different UKB ancestries *17*
Local ancestry inference by "Hapmix" 17
Joint model fitting by accounting for other environment factors 18
Using trios to conduct ancestry aware phasing for PGSs 20
References 21

Ancestry pipeline

Data Preparation

We merged the following data to generate a painting panel. From POBI[1] we extracted 2039 British samples with county-level UK annotations as described in Leslie et al. From Busby et al.[2] we extracted 511 samples with European country annotations, and a further 4077 samples from POPRES[3]. From the HGDP[4] project we extracted 1035 samples with worldwide, diverse ancestry. From Hapmap[5] we extracted 996 samples from 11 worldwide ancestry annotations. From Pagani et al.[6] we extracted 139 samples with African ancestry annotations, and a further 103 samples from Peterson et al.[7] and 189 from Schlebush et al[8]. This results in 9129 samples annotated into 4 categories: GB (shorthand for Great Britain and Ireland), Europe (Excluding GB), Africa, World (excluding World and Africa). These were available at a subset of all 2,011,414 SNPs based on their genotyping chip, to form a "union SNP list", used for imputation. Samples in the 1000Genomes[9] or UK10K[10] data were omitted from the painting panel

Label	Samples	SNPs	Reference
HAPMAP3	1184	1,383,092	(International HapMap 3 Consortium, 2010)
HGDP	1035	638,797	(Li et al., 2008)
POBI	2039	522,767	(Leslie et al., 2015)
Busby_Europe	854	471,123	(Busby et al., 2015)
POPRES	4077	373,408	(Nelson et al., 2008)
Pagani_Africa	139	816,082	(Pagani et al., 2012)
Peterson_Africa	103	882,127	(Petersen et al., 2013)
Schlebusch_Africa	200	1,914,601	(Schlebusch et al., 2012)

We performed basic relatedness pruning with Plink[11] leading to 7775 individuals (2160, 2959,899,1757) individuals from each category. We then performed phasing[12] (using SHAPEIT2) and imputation[13] (using IMPUTE4) using the UK10K + 1000 Genomes data as a reference panel. 851,948 sites that met a minimum info score threshold of 0.9 in all chips and were not outliers in terms of their pairwise MAF concordance were included in the "draft painting panel".

We then performed chromosome painting with chromopainterv2[14] for each category of individual, using that category as donors (using an individual-level painting). In general, given a set of labelled donor groups chromopainter analyses a sample and produces a “copying vector” measuring the proportion of that samples’ genome most similar to each labelled group, using haplotype sharing. For the World panel we also re-added European and African groups as donors. We screened these data for cryptically related individuals by removing individuals sharing too much genome as the most-recent match (“genome length” as a fraction of total in ChromoPainter), with genome fraction limits of (0.035, 0.035, 0.06, 0.05) respectively for (GB, Europe, Africa, World) to account for varying IBD patterns between groups within each region. From each pair of individuals we removed one at random, repeating this process until no individuals were highly related by this measure. This removes (12, 47, 121,57) leading to (2148, 2912,778,1700) individuals respectively.

We then formed “surrogate” and “donor” groups, beginning from sample labels and performed NNLS admixture estimation for each group[15], as described previously. Conceptually “donor” groups form a labelled reference panel, used for constructing copying vectors. Then, “surrogate” individuals from labelled group often (but not always; see below) matching these donor groups are used to construct summary statistics comprising their chromopainter copying vectors, which are then averaged over individuals from the same surrogate group. (Surrogate individuals must not be included in the donor group to avoid excess copying among relatives; in practice, we can achieve this by “leaving out” this individual from the corresponding donor group[14]) This results in one vector of copying proportions for each surrogate group, precisely capturing their average relatedness to each donor group. Finally, to analyse a sample we run chromopainter against the same set of donors, and fit their resulting copying vector (via NNLS) as a mixture of those constructed for the surrogates, decomposing their ancestry as some set of proportions of these labelled surrogate groups.

To construct our donor and surrogate groups, we impose that all groups are larger than (10,5,5,5) for each category, respectively (10 individuals are required within GB because genetic differences are more subtle), and that all individuals assigned to such a group must have an ancestry proportion from that group, as inferred by NNLS, of at least 50%. Then, we

iteratively (requiring only two iterations for convergence) used the following algorithm to identify initially identical donor/surrogate groups:

a) Attempt to split each group using Mclust [15]on their chunklengths, considering up to 5 clusters and proposing clusters that are the maximum *a posteriori* number of groups. We seek splits that maximally separate the samples, exceed the size restriction, and for every individual their recovery in an updated NNLS model is >0.5. In practice this procedure split only very few groups, notably Switzerland, Germany, Italy, and India.
b) Moved individuals whose preferred group was not their surrogate group to their preferred group (i.e. any group where their inferred ancestry was >50%), and remove individuals as surrogates who match no group (for example, because they possess mixed ancestry relative to the other samples).
c) Removed surrogate groups below the size threshold.
d) Where individuals needing to be removed due to steps b-c were in our "removed relatives" list, we checked whether their relatives could now be included instead according to the same criteria.

Finally we checked that surrogate groups remained consistent with their labels, removing groups that had fewer than half their samples from their originally labelled groups. Split groups were assigned new labels based on their genetic affinity to neighbouring groups.

There were a number of instances where we noticed that the above procedure failed to capture important genetic diversity. This occurs when the initial group contains only individuals that our algorithm assigned as relatives, specifically for the following pairs of groups: Native American (Surui and Pima), as well as Lahu and Melanesia, and San and Mbuti. In this case we took the paired groups and used one group (including potential relatives) as a donor, included in the painting panel, and the other as a surrogate group that we used only to estimate the expected "copying proportions". In this casethe painting should capture groups similar to the surrogate/donor pair. We were also able to use some, potentially admixed individuals as donors but not as surrogates. In Europe this was true of Irish Canadians, whilst in Africa this was true of the "Coloured" label in South Africa, as well as some ASW samples.

This leads to 6632 surrogates (1743, 2648, 752, 1489 respectively) and 6683 donors (1743, 2672, 842, 1426 respectively). We then checked the per-locus painting for these individuals and removed SNPs that showed bias by SNP chip. Specifically we removed SNPs for which the inferred mutation rate (i.e. the mismatch rate to the most similar inferred haplotype, as estimated by the chromopainter miscopying parameter) conditional on the painting was >0.2%. We then checked bias by SNP chip by taking samples with sequence data that were not in our painting or imputation panels from SGDP[16] (Mallick et al., 2016) and EGDP[17] (Pagani et al., 2016). We additionally analysed all public samples (as of November 2017) from OpenSNP (https://opensnp.org/) where individuals had been genotyped on multiple SNP chips or had released sequence data. We then downsampled all sequence data to the list of SNPs for all SNP chips and put them through the phasing and imputation pipeline. Finally, we removed all SNPs that had an imputation discordance rate of >1%. The chips were HAPMAP3, HGDP, POBI, Busby_Europe, POPRES, Pagani_Africa, Peterson_Africa, and Schlebush_Africa, UK Biobank, Ancestry v1, 23andMe v3, 23andMe v4, GSA. This resulted in 677173 SNPs forming the "final painting panel", for which imputation and phasing results were essentially identical regardless of which SNP chip was used for inference.

**Dataset**	**# samples**	**# snps**
UK Biobank (Bycroft et al., 2018)	~500k	670,741
FTDNA	13	724,620
23andMe v3	555	937,812
23andMe v4	1,043	634,617
Ancestry v1	176	676,010
GSA (Illumina)	686	588,743

We then repeated the chromosome painting with the new donor set, and repeat the admixture analysis with these groupings and performed a final iteration of steps b-c in order to ensure that our groups were robust to the exact choice of donor set.

We finally painted UK Biobank individuals who were both in regions of the world that our reference individuals capture poorly, and who have the appropriate ethnicity. Where these groups provided a strong cluster (>50% ancestry recovery for all samples and for a large enough sample as required above) we added these as surrogate by constructing a painting vector summarising typical properties of individuals from these regions. Note however that

no UK Biobank samples are used in the running of the pipeline, i.e. as donors. Instead, the surrogate vector provides summary statistics capturing what a typical person with ancestry from Netherlands, Denmark, France, Belgium, Finland, Sweden, Austria, Hundary, Ireland, Algeria, Egypt, Morocco, Libya, Nepal, Myanmar, Bangladesh, Thailand, Vietnam, Philippines, South Korea, Senegal, Liberia, Burundi, Gambia, Cameroon, Angola, Eritrea, Ethiopia, Afghanistan, Somalia, Sudan, Congo, Zambia, Ghana, Zimbabwe and Nigeria all look like in our pipeline. For reporting, we sum ancestry from the 206 surrogate groups where no clear label could be given into the 127 interpretable ancestry labels reported in our results section.

Annotating population labels into genetic groupings is an art, not a science and reasonable people may have made different decisions based on preferences towards "lumping or splitting" similar groups on a genetic continuum and a need to make ancestry components interpretable via a map. For replicability we therefore include all annotations necessary for this process in the online supplementary materials; specifically the 3 sets of SNP lists, and the donor and surrogate group annotations.

References

1. Leslie, S. *et al.* The fine-scale genetic structure of the British population. *Nature* **519**, 309-314 (2015).
2. Busby, G.B. *et al.* The role of recent admixture in forming the contemporary West Eurasian genomic landscape. *Current Biology* **25**, 2518-2526 (2015).
3. Nelson, M.R. *et al.* The Population Reference Sample, POPRES: a resource for population, disease, and pharmacological genetics research. *The American Journal of Human Genetics* **83**, 347-358 (2008).
4. Li, J.Z. *et al.* Worldwide human relationships inferred from genome-wide patterns of variation. *science* **319**, 1100-1104 (2008).
5. Consortium, I.H. Integrating common and rare genetic variation in diverse human populations. *Nature* **467**, 52 (2010).
6. Pagani, L. *et al.* Ethiopian genetic diversity reveals linguistic stratification and complex influences on the Ethiopian gene pool. *The American journal of Human genetics* **91**, 83-96 (2012).
7. Petersen, D.C. *et al.* Complex patterns of genomic admixture within southern Africa. *PLoS genetics* **9**, e1003309 (2013).
8. Schlebusch, C.M. *et al.* Genomic variation in seven Khoe-San groups reveals adaptation and complex African history. *Science* **338**, 374-379 (2012).
9. Consortium, G.P. A global reference for human genetic variation. *Nature* **526**, 68 (2015).

10. Walter, K. *et al.* The UK10K project identifies rare variants in health and disease. *Nature* **526**, 82-90 (2015).
11. Chang, C.C. *et al.* Second-generation PLINK: rising to the challenge of larger and richer datasets. *Gigascience* **4**, s13742-015-0047-8 (2015).
12. Delaneau, O., Zagury, J.-F. & Marchini, J. Improved whole-chromosome phasing for disease and population genetic studies. *Nature methods* **10**, 5 (2012).
13. Bycroft, C. *et al.* The UK Biobank resource with deep phenotyping and genomic data. *Nature* **562**, 203-209 (2018).
14. Lawson, D.J., Hellenthal, G., Myers, S. & Falush, D. Inference of population structure using dense haplotype data. *PLoS genetics* **8**, e1002453 (2012).
15. Scrucca, L., Fop, M., Murphy, T.B. & Raftery, A.E. mclust 5: clustering, classification and density estimation using Gaussian finite mixture models. *The R journal* **8**, 289 (2016).
16. Mallick, S. *et al.* The Simons genome diversity project: 300 genomes from 142 diverse populations. *Nature* **538**, 201-206 (2016).
17. Pagani, L. *et al.* Genomic analyses inform on migration events during the peopling of Eurasia. *Nature* **538**, 238-242 (2016).

Phenotypes in the UKB

We selected 104 representative quantitative phenotypes in the UK Biobank to conduct different analyses. Most of the phenotypes are directly pulled out from the UKB raw data, except the following phenotypes defined algorithmically: Education attainment score (EA), Systolic blood pressure (SBP), Diastolic blood pressure (DBP), pulse pressure (PP) and Estimated glomerular filtration rate (eGFR).

Definition of EA

The definition of Education attainment score is based on the UKB field 6138 "Qualification". We use the following criterion to convert categorical variable to quantitative variable:

For participants self-reported as "prefer not to answer", we assigned NA. For the rest of participants, we assigned the score based on the following International Standard Classification for Education (ISCED) definition[1,2]:

$$ES = \begin{cases} 7, if \text{ "none of the above"} \\ 10, if \text{ "CSEs or equivalent" or "O levels/GCSEs or equivalent"} \\ 13, \text{"A levels/AS levels or equivalent"} \\ 15, \text{Other professional qualifications eg: nursing, teaching} \\ 19, \text{NVQ or HND or HNC or equivalent} \\ 20, \text{College or University degree} \end{cases}$$

Definition of SBP, DBP and PP

The definition of systolic blood pressure and diastolic blood pressure is based on previous literature[3,4].

The mean SBP and DBP were calculated by using automated blood pressure readings from the first assessment visit (UKBB fields 4080 and 4079) and adjusted for medication usage by adding 15 mmHg and 10 mmHg respectively. If the automated reading was not available, manual measurements (UKBB fields 93 and 94) were used. We further calibrated the blood pressure by setting the readings to missing if the value is more than four standard deviations from the mean. PP was then calculated as SBP minus DBP.

Definition of eGFR

eGFR was defined by using four UKBB fields: Creatine (UKBB filed 30700), age at recruiment (UKBB field 21022), sex (UKBB field 31) and Ethnic background (UKBB field 21000). Participants were defined black ethnicity if their ethnic background codes are 4001 (Caribbean), 4002 (African), 4003 (Any other black background). The eGFR was then derived by "CKDEpi.creat" function in R package "nephron" (https://cran.r-project.org/package=nephro).

References

1. Okbay, A. *et al.* Genome-wide association study identifies 74 loci associated with educational attainment. *Nature* **533**, 539-542 (2016).
2. Carter, A.R. *et al.* Educational attainment as a modifier for the effect of polygenic scores for cardiovascular risk factors: cross-sectional and prospective analysis of UK Biobank. *International Journal of Epidemiology* **51**, 885-897 (2022).
3. Tobin, M.D., Sheehan, N.A., Scurrah, K.J. & Burton, P.R. Adjusting for treatment effects in studies of quantitative traits: antihypertensive therapy and systolic blood pressure. *Statistics in medicine* **24**, 2911-2935 (2005).
4. Kerin, M. & Marchini, J. Inferring gene-by-environment interactions with a Bayesian whole-genome regression model. *The American Journal of Human Genetics* **107**, 698-713 (2020).

Genetic ancestry in the UKB

Visualisation of the inferred genetic ancestry/entropy of UK-born UKB samples

To visualise variation in genetic ancestry, or the entropy of genetic ancestry, across the UK, we used a Gaussian kernel smoothing technique. The genetic ancestry is averaged at each pixel weighted by the Gaussian kernel:

$$\bar{O} = \sum_{i=1}^{N} \frac{e^{-q((x_p-x_i)^2+(y_p-y_i)^2)}}{\sum_{i=1}^{N} e^{-q((x_p-x_i)^2+(y_p-y_i)^2)}} O_i$$

where $\bar{O}$ is the average value (of genetic ancestry or entropy) assigned to a given pixel, O_i is the value for individual *i* ($1 \le i \le N$), N=426,879 is the number of UK-born or Ireland born UKB participants, and (x_p,y_p) and (x_i,y_i) ($1 \le i \le N$) are coordinates of the pixel centre and the birthplace of the UKB individual *i* respectively. The weight *q* is pixel-specific and determined by adaptive bandwidth smoothing as follows, so as to keep the "effective" number of individuals per pixel approximately constant at 200:

$$S = \sum_{i=1}^{N} e^{-q((x_p-x_i)^2+(y_p-y_i)^2)}$$

Repeat

q=q/2

$$S = \sum_{i=1}^{N} e^{-q((x_p-x_i)^2+(y_p-y_i)^2)}$$

Until S<200

Return *q*

Summary of entropy of UKB participants born in the UK/Ireland

For each participant born in the UK or Republic of Ireland, we calculated an entropy *E* (Methods) to quantify their degree of mixed ancestry (note, this is relative to our assigned group labels). We observed that overall, major cities, and their surrounds, show more mixing on average, possibly reflecting historical migrations to these places. The highest entropy in the UK is for individuals born in London, which also possesses the highest average fraction of ancestry from elsewhere in the world. West Yorkshire has the lowest value of *E,* while lower values are seen for more mountainous and/or remote parts of the UK in general, suggesting that migration into these regions has left less genetic impact. We also note that Oxfordshire

has an interesting signal: a moderate value of *E*, but much lower than the surrounding areas, and the barrier of *E* around Oxfordshire almost coincides the boundary of Oxfordshire (Extended Data Fig.2).Because some of our donor samples[1] are from Oxfordshire, it is unclear whether this reflects a genuine reduction of mixture relative to surrounding areas or simply more continuous population structure, but regardless, it indicates that county-scale ancestry differences can occur even in regions were previous population structure was not apparent[1].

Reference

1. Leslie, S. *et al.* The fine-scale genetic structure of the British population. *Nature* **519**, 309-314 (2015).

Estimation of regional allele frequencies in the UK and worldwide

Expectation-maximization based method to estimate regional allele frequency

Highly differentiated alleles across different regions may indicate strong local genetic drift, or act as a signature of natural selection. We used an expectation-maximization (EM) algorithm to estimate regional allele frequencies based on ancestral inference for each individual and region (Methods). We ran the EM on 25,485,700 imputed UKB SNPs, and estimate their allele frequencies across the 23 British-Irish regions, and in 83 worldwide regions, using two different UKB groups (see below).

Using the ancestral inference for each individual, we developed an EM algorithm to infer the regional allele frequencies. We constructed a $2N_s \times N_m$ haplotype matrix by splitting each genotype into two single haplotypes, where N_s is the number of individuals and N_m is the number of SNPs of which the allele frequency will be estimated. In the *n*th EM iteration, the expectation of h_{ijm}, the number of copies of the *i*th haplotype derived from ancestral region *j* at locus *m*, is calculated by (using Bayes theorem):

$$E\left(h_{ijm}\right)^{(n)} = \frac{P\left(h_{ijm}\middle|i \in I_j\right)P(i \in I_j)}{\sum_{j=1}^{N_a} P\left(h_{ijm}\middle|i \in I_j\right)P(i \in I_j)} \tag{1}$$

where N_a is the total number of ancestral regions, and I_j is the set of individual haplotype from *j*th group. The probability $P(i \in I_j)$ that individual (haplotype) *i* is from the *j*th group is equal to the ancestry coefficient a_{ij} inferred from our ancestry pipeline: we assume that conditional on local ancestry being drawn from group *j*, h_{ijm} follows a Bernoulli distribution with parameter f_{jm} , where f_{jm} is the allele frequency of the SNP at the *m*th locus in region *j*. It is these frequencies that we seek to infer. These assumptions lead us on to:

$$E\left(h_{ijm}\right)^{(n)} = \frac{f_{jm(n)}^{h_{ijm}}\left(1-f_{jm(n)}\right)^{1-h_{ijm}}a_{ij}}{\sum_{j=1}^{N_a} f_{jm(n)}^{h_{ijm}}\left(1-f_{jm(n)}\right)^{1-h_{ijm}}a_{ij}}. \tag{2}$$

We then update the allele frequency f_{jm} in the maximization step using the following step (this maximises the full data likelihood where local ancestry is fully known):

$$f_{jm(n+1)} = \frac{\sum_{i=1}^{2N_s} E\left(h_{ijm}\right)^{(n)} I(h_m = 1)}{\sum_{i=1}^{2N_s} E\left(h_{ijm}\right)^{(n)}} \tag{3}$$

where $I(h_m=1)$ is the indicator variable of the mutation type "1" being present on this chromosome at this SNP. We iterate (2) and (3) until convergence is reached, as defined by the frequency reaching stability:

$$\left\|\boldsymbol{f}_{j(n+1)} - \boldsymbol{f}_{j(n)}\right\| < \delta \quad (4)$$

where $\boldsymbol{f}_{j(n)} = (f_{1(n)}, \dots, f_{N_m(n)})$ and "||.||" is the Euclidean norm. We set δ as 0.001 in our analysis.

We applied this EM to those UKB SNPs with minor allele frequency (MAF) >0.0001 and information score >0.9. We applied the EM in two different subsets of UKB samples, with one subset including all individuals with at least 95% inferred UK+Ireland ancestry, and the remaining samples forming another subset. We call the former subset the "UK+Ireland" subset and the latter the "world subset". For the UK+Ireland subset, we only rescaled the genetic ancestry of the participants such that the sum of their 23 UK+Ireland ancestries equals 1. These rescaled ancestries were then used as input for the EM algorithm to estimate the regional allele frequencies of each SNP in each of the 23 UK + Ireland regions.

For the world subset, we removed all regions with mean ancestry less than 1%, and rescaled the remaining regions such that they again sum up to 1. This resulted in 82 remaining regions whose allele frequencies were estimated in the world subset.

Variant rs5743618

A large number of variants exhibiting varying allele frequency across UK were identified. One such is rs5743618 discussed in the main text that has been strongly associated with hay-fever in previous GWAS of European populations. The allele frequency of this variant shows a clear decline from south to north, with the maximum allele frequency difference 24%.

GWAS analysis

Running BOLT-LMM for place of birth (North/South)

BOLT-LMM[1] is a linear mixed model based method which efficiently corrects the population structure and cryptic relatedness in the samples. We applied BOLT-LMM for the trait "Place of birth in UK -North coordinate"(UKB field 129), a trait for which PC-corrected GWAS yields substantial false positives.

We downloaded the autosome genotype call data (UKB field ID 22418) from UKB, and pulled out the same 343,047 white British samples that we used for running AC-corrected and PC-corrected GWAS. We used "*plink1.9*"[2] to conduct variant quality control by filtering out variants in high LD regions, with minor allele frequency less than 0.01, genotype missingness less than 0.05 and Hardy-Weinberg equilibrium p-value less than 1×10^{-6}. Afterwards, we used "*plink1.9*" to run LD-pruning with option "--indep-pairwise 1000 100 0.1". Finally, we got in total 142,182 independent chip array SNPs on autosomes, as input for both the "*flashpca*2"[3] and null model building for BOLT-LMM.

20PCs output from "*flashpca*2" together with genotype batch, age, sex, age^2, agexsex and age^{2}xsex were used as covariates in the BOLT-LMM analysis. In BOLT-LMM, we also set parameters "bgenMinMAF=0.001" and "bgenMinINFO=03" in line with the setting we ran for "bgenie". "P-BOLT-LMM" column from the output of BOLT-LMM was used to obtain the p-value of BOLT-LMM in the downstream analysis.

GWAS with different number of PCs

For the 104 traits we ran AC-corrected GWAS and PC-corrected GWAS using both 20, and 40, PCs while keeping the other covariates the same. After thinning the resulting genome-wide significant variants within 200kb to keep only the most significant variant in each region, we then compared the p-value of independent variants when using 20 or 40 PCs. 48 among the 99 traits possess at least one variant with a p-value change between the 20PC and 40 PC-corrected GWAS that is greater than 100 fold, as summarized in the following table:

Traits	#variants |log10(P(pc40)/P(pc20)|>2
Calcium	2
Cystatin C	1
eGFR	2
Gamma glutamyltransferase	1
IGF1	3
PP	3
SBP	5
SHBG	1
Triglycerides	1
Urate	1
Whradjbmi	1
Place of birth in UK north co ordinate	17
Place of birth in UK east co ordinate	14
Sitting height	10
Home location at assessment east coordinate rounded	7
Home location at assessment north coordinate rounded	7
Neuroticism score	1
Body mass index BMI	7
Weight	2
Home location east coordinate rounded	6
Home location north coordinate rounded	9
Body fat percentage	10
Whole body fat mass	8
Leg fat percentage right	10
Leg fat mass right	9
Leg fat percentage left	14
Leg fat mass left	5
Arm fat percentage right	5
Arm fat mass right	1

Arm fat free mass right	1
Arm fat percentage left	7
Arm fat mass left	1
Arm fat free mass left	1
Trunk fat percentage	9
Trunk fat mass	8
Trunk fat free mass	1
Employment score England	12
Education score England	5
Housing score England	1
Forced vital capacity FVC	4
Forced expiratory volume in second FEV	6
Peak expiratory flow PEF	4
Ankle spacing width	9
Heel bone mineral density BMD	2
Ankle spacing width left	5
Ankle spacing width right	6
Hip circumference	3
Standing height	27

References

1. Loh, P.-R. *et al.* Efficient Bayesian mixed-model analysis increases association power in large cohorts. *Nature genetics* **47**, 284-290 (2015).
2. Chang, C.C. *et al.* Second-generation PLINK: rising to the challenge of larger and richer datasets. *Gigascience* **4**, s13742-015-0047-8 (2015).
3. Abraham, G., Qiu, Y. & Inouye, M. FlashPCA2: principal component analysis of Biobank-scale genotype datasets. *Bioinformatics* (2017).

Portability of polygenic score across different UKB ancestries

Local ancestry inference by "Hapmix"

In order to get the local PRS deconvolution, we selected the 8003 UKB European-African admixed individuals with the sum of (27 UK/European ancestries and 4 African ancestries) larger than 90%. We estimated the local ancestry of each Hapmap3 SNP for each individual using "hapmix"[1] with the default parameter setting. 1000G phase 3 CEU and YRI were chosen as two surrogates representing the European and African ancestries in the reference panel. Output from "hapmix" codes, for each SNP, the joint probability $h(P_1, h_1, P_2, h_2) = Prob(P_1, h_1, P_2, h_2)$ in the *k*th column, where $k = P_1 \times 2^3 + h_1 \times 2^2 + P_2 \times 2^2 + h_1 \times 2^0 + 1$. P_1 and P_2 are indicator variables that equal 0 (1) if that SNP possesses European (African) ancestry at the first (P_1) and second (P_2) chromosomal copy (haplotype), and h_1 and h_2 are also indicator variables now encoding whether the allelic type of this SNP is 1 or 0 at the first and second haplotypes. For the SNP at the *j*th locus for the *i*th individual, G_{ij}^{EE} is the expected "1" allele count, occurring on a background where both haplotype ancestries are European; G_{ij}^{EA} is the expected "1" allele count on the European haplotype, occurring on a background where one of the haplotypes is European and the other is African; Similarly, we defined G_{ij}^{AA} and G_{ij}^{AE} as the expected "1" allele counts when both haplotypes are African, or on the African haplotype when the other ancestral haplotype is European (note that G_{ij}^{EA} and G_{ij}^{AE} are different, reflective of ancestry-specific haplotypic phasing by hapmix). G_{ij}^{EE}, G_{ij}^{EA}, G_{ij}^{AA} and G_{ij}^{AE} are calculated by summing "hapmix" probabilities as follows:

G_{ij}^{EE}= h(0,1,0,0)+h(0,0,0,1)+h(0,1,0,1)+h(0,1,0,1)

G_{ij}^{EA}= h(0,1,1,0)+h(0,1,1,1)+h(1,0,0,1)+h(1,1,0,1)

G_{ij}^{AA}= h(1,1,1,0)+h(1,0,1,1)+h(1,1,1,1)+h(1,1,1,1)

G_{ij}^{AE}= h(1,1,0,0)+h(1,1,0,1)+h(0,0,1,1)+h(0,1,1,1).

We also calculated P_{ij}^{EE}, the probability that both haplotypes have European ancestry, $P_{ij}^{E\backslash A}$, the probability that one haplotype is European and another is African, and P_{ij}^{AA} as the probability that both haplotypes are African, based on the hapmix output.

Finally, total local European ancestry A_{ij}^{E} at the *j*th locus for the *i*th indvidual was calculated as follows: Let C_{ij}^{E} is the number of copies from European ancestry at *j*th locus,

$$A_{ij}^{E} = P_{ij}^{E\backslash A} + 2\, P_{ij}^{EE}.$$

We observed highly consistent genome-wide (average) inference of African ancestry from both "hapmix" and the ancestry pipeline:

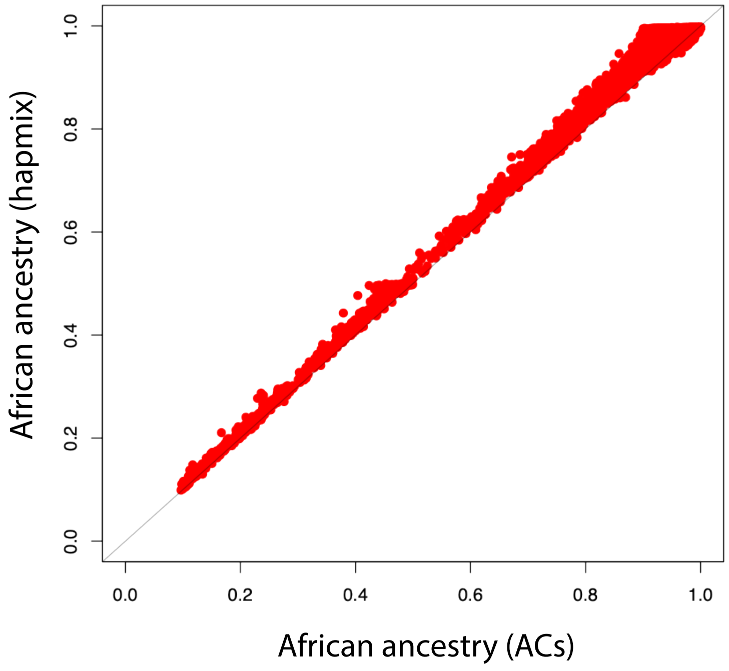

Supplementary Fig. 19. Comparison of inferred African ancestry obtained by ACs (x-axis) and by Hapmix (y-axis)

Joint model fitting by accounting for other environment factors

We further investigate if other environment factors, such as birth country or gender, have impact on the portability of polygenic scores. Based on the model in the main text (Methods), we extend the model by adding a new parameter which indicates if a sample individual was born in UK, and fit the model in male and female samples separately. The extended model is as follows:

$$Y_i = I + \left(\beta_{Eu}\mathrm{EPGS}_i + \beta_{Af}\mathrm{APGS}_i\right) \times (1 + \omega\theta_i + \varphi\zeta_i) + covariates + \varepsilon_i \qquad (1)$$

where for individuals $i = 1,2, \ldots n$, Y_i is the phenotype, ε_i is the zero-mean noise, and the parameters to be estimated are the intercept *I*, and $\beta_{Eu}, \beta_{Af}, \omega, \varphi$. EPGS_i and APGS_i are the centralized EPGS and APGS as above, now after regressing out the covariates, and θ_i is the mean genome-wide European ancestry proportion and ζ_i is the indicator variable that whether the sample was born in UK (1 if born in UK, 0 otherwise) for individual $i = 1,2, \ldots n$.

Based on the model above, we can estimate the effect size of European PGS or African PGS with varying global ancestry for people born in UK or born outside. By stratifying the populations in terms of their gender, we fit this extended model in male and female samples separately (Extended Data Fig. 10) in three scenarios: 1). $\omega \neq 0$ and $\varphi = 0$; 2). $\omega = 0$ and $\varphi \neq 0$; 3). $\omega \neq 0$ and $\varphi \neq 0$. Supplementary Fig. 20 shows the distribution of genetic ancestry stratified by gender and birth places:

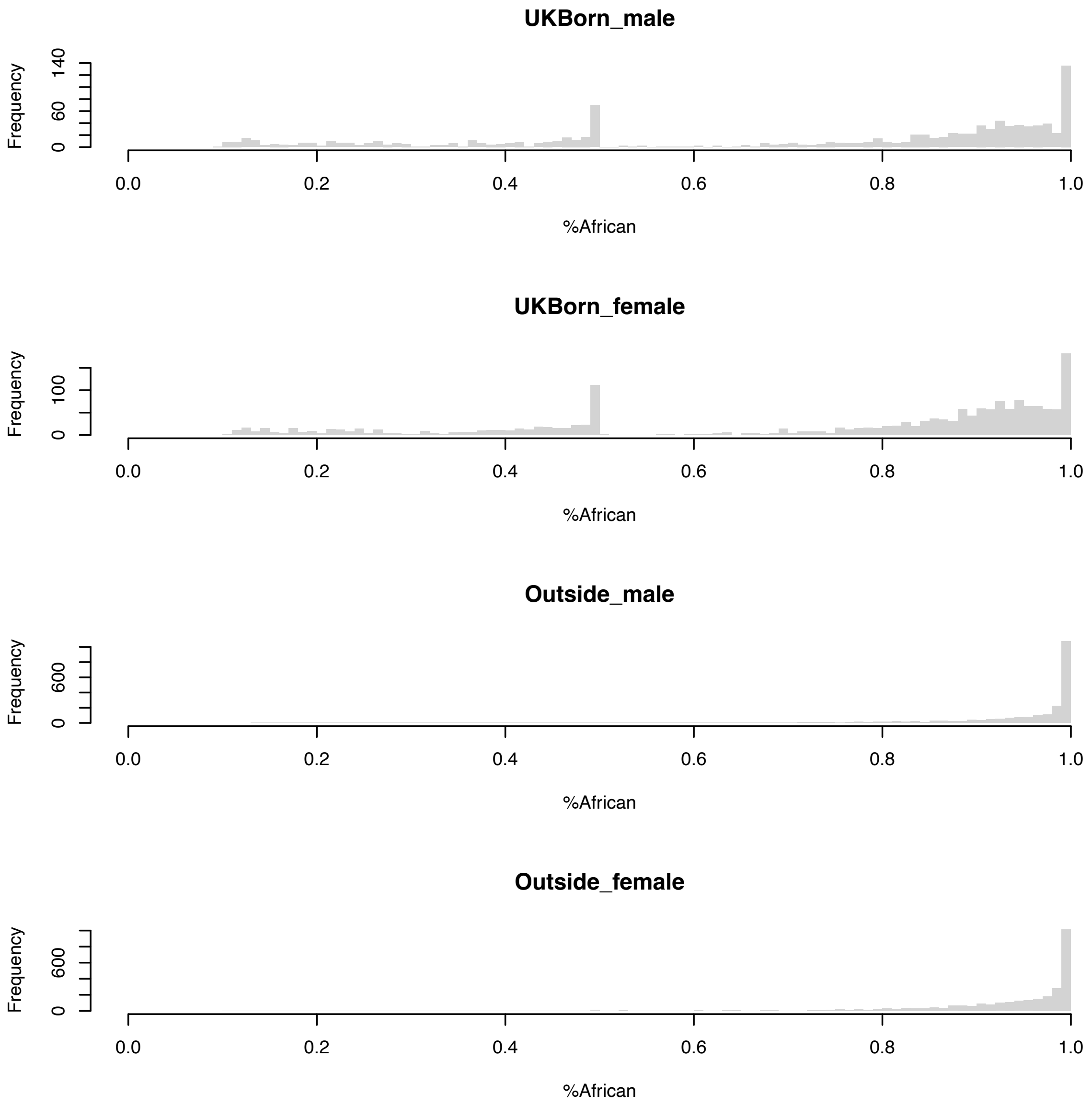

Supplementary Fig. 20. Distribution of African ancestry stratified by gender and whether individuals are born within the UK, or outside the UK.

Using trios to conduct ancestry aware phasing for PGSs

We extracted the genotypes of African-ancestry individuals from the raw chip genotype data and conducted quality control by removing the SNPs in regions of high LD, with missing rate larger than 0.05 and with Hardy-Weinberg p-value less than 0.0000001. Using "king"(version 2.2.4)[2] we then identified two trios among the 8003 African-ancestry individuals. For the identified trios, we reran Hapmix with phase specified: heterozygous sites in children are encoded as as "3" if the allele "1" is paternal and "4" if the allele "1" is maternal, and for

parents un-transmitted heterozygous allele “1” cases are recoded as “3” and transmitted sites are recoded as “4”.

In trio individuals this results in direct haplotype-based “phased” estimation of the European PGS (EPGS) and African (APGS) of trio individuals across 53 phenotypes, which we compared to their unphased equivalents in the same individuals which were calculated as for the remainder of the 8003 African-ancestry people. To enable joint analysis, we normalised the resulting phased and unphased EPGS and APGS in the following way. Let $\boldsymbol{U}$ be the column vector of unphased EPGS or APGS and $\boldsymbol{P}$ the column vector of phased EPGS or APGS, and $\boldsymbol{C}$ the matrix with **1, age, sex and 31 ACs** as columns. Then we fit the model:

$$\boldsymbol{U} = \boldsymbol{C\beta} + \boldsymbol{\varepsilon}$$

where $\boldsymbol{\varepsilon}$ is the residual. If $\widehat{\boldsymbol{\beta}}$ is the least square estimator of $\boldsymbol{\beta}$, we obtain the residual for both $\boldsymbol{U}$ and $\boldsymbol{P}$:

$$\hat{\boldsymbol{\varepsilon}}_{\boldsymbol{U}} = \boldsymbol{U} - \boldsymbol{C}\widehat{\boldsymbol{\beta}}$$

$$\hat{\boldsymbol{\varepsilon}}_{\boldsymbol{P}} = \boldsymbol{P} - \boldsymbol{C}\widehat{\boldsymbol{\beta}}$$

We then get the mean and variance of residual of $\boldsymbol{U}$ weighted by individual European ancestry $\boldsymbol{A}_E$ for unphased EPGS or African ancestry $\boldsymbol{A}_F = 1 - \boldsymbol{A}_E$ for unphased APRS.

$$\bar{\varepsilon}_U = \frac{\hat{\boldsymbol{\varepsilon}}_{\boldsymbol{U}}^{\boldsymbol{T}} \boldsymbol{A}_E}{\boldsymbol{1}^{\boldsymbol{T}} \boldsymbol{A}_E}$$

$$var(\hat{\boldsymbol{\varepsilon}}_{\boldsymbol{U}}) = \frac{(\hat{\boldsymbol{\varepsilon}}_{\boldsymbol{U}} - \bar{\varepsilon}_U \boldsymbol{A}_E)^{\boldsymbol{T}} (\hat{\boldsymbol{\varepsilon}}_{\boldsymbol{U}} - \bar{\varepsilon}_U \boldsymbol{A}_E)}{\boldsymbol{1}^{\boldsymbol{T}} \boldsymbol{A}_E}$$

For trio individuals, the normalized $\boldsymbol{U}$ and $\boldsymbol{P}$ can be obtained as follows:

$$\widehat{\boldsymbol{U}} = \frac{\hat{\boldsymbol{\varepsilon}}_{\boldsymbol{U}} - \bar{\varepsilon}_{\boldsymbol{U}} \boldsymbol{A}_E}{\sqrt{var(\hat{\boldsymbol{\varepsilon}}_{\boldsymbol{U}})}}$$

$$\widehat{\boldsymbol{P}} = \frac{\hat{\boldsymbol{\varepsilon}}_{\boldsymbol{P}} - \bar{\varepsilon}_{\boldsymbol{U}} \boldsymbol{A}_E}{\sqrt{var(\hat{\boldsymbol{\varepsilon}}_{\boldsymbol{U}})}}.$$

We used these normalised scores to compare across different phenotypes.

References

1. Price, A.L. *et al.* Sensitive detection of chromosomal segments of distinct ancestry in admixed populations. *PLoS genetics* **5**(2009).
2. Manichaikul, A. *et al.* Robust relationship inference in genome-wide association studies. *Bioinformatics* **26**, 2867-2873 (2010).

Supplementary Note 2
Estimation of polygenic score (PGS) coefficients in admixed individuals

Notation and assumptions

We will use the indexes i to denote an individual in a sample of size N, and j, k to index a polymorphism (SNP) within an individual, among S SNPs in total which we will assume to be linearly ordered along the genome. For simplicity, we will consider two ancestries labelled as A and E; it is not necessary that these represent "European" and "African" regional ancestries, but in our main application setting this is the case. We will assume we may regard these populations as relatively homogeneous (at least relative to the genetic distinctness of each relative to the other). We also consider the diploid genome as two contiguous haploid stretches, so that the "genotype" at SNP j is either 0 or 1, and there is only one ancestry at each position. Because we are assuming a linear polygenic score in this section, this has no impact on our results, except there are two copies of each SNP coefficient (with identical effect sizes), corresponding to the two chromosomal copies. We assume Hardy-Weinberg equilibrium, so the genotypes on these two chromosomal copies are independent, and their ancestries are also independent given the appropriate genome-wide ancestry for an individual.

When there is admixture, ancestry in general varies along the genome and across individuals. Individual i has genome-wide population A mean ancestry θ_i. At SNP j, define an indicator variable I_{ij} which is 1 if individual i possesses ancestry drawn from A at SNP j and 0 if their ancestry is instead E at SNP j; we will sometimes neglect the i subscript while considering a single individual.

The genotype of an individual at a particular SNP is denoted by g_{ij}. We will also utilise mean genotypes at SNP j; these are given by $\bar{g}_j^A$ and $\bar{g}_j^E$ to represent the mean frequencies in populations E and A respectively. It will also be helpful to consider the mean genotype of individual i at SNP j; this is denoted by $\bar{g}_{ij} = \theta_i \bar{g}_j^A + (1 - \theta_i)\bar{g}_j^E$.

We will denote the confounders/coregressors for individual i as a vector C_i (forming a matrix C across individuals) whose first entry $C_{i1} = \theta_i$, the ancestry. Let the observed phenotype value for individual i be Y_i.

SNPs impact the trait of interest. As is standard, we will assume linear additive effects across SNPs such that SNP j has true effect γ_j (many authors use β_j for the effect size, but we use γ_j simply to avoid a clash of notation). We will *not* require that these effects have any particular distribution, other than that the trait is polygenic. We also assume that one or more genome-wide association studies (GWAS) has been conducted, resulting in an estimated effect size $\hat{\gamma}_j$ for each SNP j. We will assume that individual i (or their relatives) were not included in these GWAS, so we may regard this estimate as independent of the genetic data of this individual (this is true for our real-data applications).

We will additionally make two further simplifying assumptions about the use of PGS. First, we will assume coregressors (or confounders) corrected for in the estimation procedure (e.g. age, gender, genotyping centre, etc) are independent of genotypes featuring in the PGS, conditional on genome-wide ancestry; we will

assume that, as is standard in genome-wide association studies, genome-wide ancestry is also corrected for as a confounder. Second, we will assume ancestry segments within individual are "large" in the following sense: if two SNPs are in different ancestry segments, then we may assume no linkage disequilibrium (LD) between them in either founder population, or across founder populations. Informally this assumption means that ancestry segments extend further than LD, and it is likely to be reasonably well satisfied for groups admixing within the past few hundred generations.

Overview of approach

The heart of the ANCHOR approach is to consider different groups of non-admixed and admixed individuals (for example stratified by ancestry proportion, or combined), that share some ancestry E and also possess a second ancestry A in varying proportions.

For each group we identify those segments of the genome of each ancestry A and E and use a previous GWAS, conducted for example in an independent sample of ancestry E, to generate two polygenic scores (PGS) $EPGS$ and $APGS$ for each individual, respectively using only genotypes in the A and E segments of that individual's genome, and then fit the regression

$$Y \sim EPGS + APGS + coregressors.$$

In this Supplementary Note we will show that the inferred coefficients for $EPGS$ and $APGS$ typically differ from each other, but that provided we build these scores appropriately, their true values are the same across groups if the effect sizes γ_j are shared. Alternatively, effect sizes differ among the groups considered, indicating gene-environment or gene-gene interactions. In this case, we will show that the $EPGS$ and $APGS$ coefficients allow quantification of group differences - e.g. estimation of the correlation in underlying effect sizes - among the groups considered.

We will also show (and demonstrate using simulations) that there is a "correct" way to construct $EPGS$ and $APGS$, mean-centring genotypes conditional on local ancestry, while other constructions cause biases. Although the explanation is somewhat technical, in brief mean-centring is necessary in practice because three phenomena interact: first, underlying "true" effect sizes operating in the admixed population are not known. Secondly, SNP allele frequencies differ between admixing populations. Thirdly, ancestry varies randomly along the genome in admixed individuals. If any of those three were not true, mean-centring would not be needed, but in practice all occur, in almost all real-world applications. When they do occur, the average value of the phenotype estimate from the PGS when applied to admixed individuals depends on their random local ancestry, and mean SNP frequencies in E and A. Because effect sizes are only estimates, this adds additional ancestry-dependent noise to phenotype prediction, and we show below this noise biases the resulting the $EPGS$ and $APGS$ coefficients, in theory and in practice.

Models and model fitting

To begin to derive the ANCHOR procedure, for our "true" underlying model, we consider the setting where our phenotype

$$Y_i = \alpha + \sum_{j=1}^{S} \gamma_j g_{ij} + \mu C_i + \epsilon_i, \tag{1}$$

where α and the vector μ are coefficients expressing the impacts of a constant term, and the confounders, on the phenotype for individual i. Here ϵ_i is the error due to e.g. randomness or environmental variability. We assume this form for the true behaviour throughout this Note. Our null model of shared effects is then simply indicating that the same terms γ_j apply identically across individuals, whatever their admixture proportions and whatever their population of origin, though the other terms may vary. The alternative is that γ_j values might differ across populations, or groups of different ancestry proportions. The vector product μC_i in the above has first term $\mu_1 \theta_i$ and typically might also include other ancestry terms (e.g. PCs), so allows for impacts of ancestry on the phenotype Y_i.

We would like to use this model, but unfortunately the values of γ_j are not known to us, and so instead in typical GWAS we replace these terms with their estimates, fitting the model:

$$Y_i = \alpha + \beta \sum_{j=1}^{S} \hat{\gamma}_j g_{ij} + \mu C_i + \epsilon_i. \tag{2}$$

Here, note that the sum in the second term is the estimated PGS. We must also introduce a term β in front of this term. This accounts for the fact that it is likely our PGS may, for example, systematically estimate effect sizes (winner's curse effect), include SNPs not truly associated with the trait, or include variants in LD, each of which will result in the variance of the PGS estimate not completely capturing variance of the true PGS. Accordingly, we expect $0 < \beta < 1$ in most cases. We also require that different individuals are approximately unrelated, so their genotypes can be regarded as independent, once their ancestries are taken into account. However, in our approach we are otherwise agnostic as to how the coefficients $\hat{\gamma}_j$ are obtained (apart from this occurring in an independent sample). Specifically, we require no particular assumptions about what approach is used to correct for LD, or penalisation of the coefficients etc - these will impact the particular value of β that is appropriate, but do not otherwise impact our results. The levels of LD within populations are also not important, apart from the assumption that ancestry segments extend further than within-population LD in admixed individuals. The below derivations only require that the above linear model (1) holds; in other words, that on average Y_i increases linearly with the estimated PGS value.

0.1 Least-squared estimators

In the standard approach to quantifying the variance explained by the PGS, the squared correlation between the phenotype and PGS is obtained by fitting the least-squares estimators $\hat{\beta}$ of β, and of the other parameters, in the model (2) and then calculating the mean squared residual variance relative to the variance when $\beta = 0$. The least squares estimator represents the mle of the parameters under the assumption that ϵ_i is normally distributed, and is unbiased and consistent provided the mean of the residuals ϵ_i are zero for each individual, and the residual variances are bounded.

Assuming our true model holds with zero-mean residuals, then if we have a combined matrix X of predictors (we can view this equation as a definition of the parameters β):

$$\mathbb{E}_{X,Y}\left(X^T Y\right) = \mathbb{E}_X(X^T X)\beta.$$

As a corollary, it can be shown that the true value of β is that value which minimises the expected mean squared residuals over individuals; or equivalently the average value for a randomly chosen individual i, $\mathbb{E}\left(\epsilon_i^2\right)$, where the expectation is taken over both the data Y and the predictors X (viewed as random within the population). Further the standard least-squares estimator of β is unbiased, and asymptotically consistent in large samples (we typically expect GWAS datasets to contain thousands of individuals).

Our approach in this Supplementary Note is to derive true parameter values in different populations by obtaining those values minimising $\mathbb{E}\left(\epsilon_i^2\right)$, in settings with shared or non-shared effect sizes. This will allow testing for shared vs. non-shared effect sizes in practice, leveraging the least-squares estimators of these parameters.

0.2 Homogeneous populations

First, we consider the simplest possible setting: a sample of individuals each drawn from population E. In this case, the true value of β is β_E say and this minimises $\mathbb{E}\left(\epsilon_i^2\right)$ under the model

$$Y_i = \alpha + \beta_E \sum_{j=1}^{S} \hat{\gamma}_j g_{ij} + \mu C_i + \epsilon_i. \tag{3}$$

Note that because the model fit includes a constant α, fitting the following model which will be more convenient yields an identical estimate of β:

$$Y_i = \alpha + \beta_E \sum_{j=1}^{S} \hat{\gamma}_j \left(g_{ij} - \bar{g}_j^E\right) + \mu C_i + \epsilon_i.$$

By the same argument, for a sample of individuals drawn from population A we have a true value β_A say which minimises $\mathbb{E}\left(\epsilon_i^2\right)$ in the model:

$$Y_i = \alpha + \beta_A \sum_{j=1}^{S} \hat{\gamma}_j \left(g_{ij} - \bar{g}_j^A\right) + \mu C_i + \epsilon_i.$$

In the setting where the coefficients $\hat{\gamma}_j$ are estimated using GWAS in populations similar to E, we expect $0 < \beta_A < \beta_E < 1$. This is because due to regional linkage disequilibrium differences the estimated PGS is expected to on average be a better model for the true PGS in populations genetically closer to those used for the discovery. We note that using e.g. $g_{ij} - \bar{g}_j^E$ in place of g_{ij} means using *mean-centred* genotypes i.e. a mean-centred PGS.

In a homogeneous population, as we have seen above the choice of whether to centre genotypes is arbitrary, and so researchers often use either form interchangeably. However, we will show using theory and simulations that the choice has important consequences in the case of an admixed population; and so we strongly recommend the use of mean-centring, conditional on *local* ancestry, in this case.

0.3 Populations incorporating admixed individuals

For this section, we will consider the setting where we now analyse admixed individuals, and assume model (1) as before, slightly reparameterised:

$$Y_i = \alpha^o + \sum_{j=1}^{S} \gamma_j \left(g_{ij} - \bar{g}_{ij}\right) + \mu^o C_i + \epsilon_i^o. \tag{4}$$

Given this underlying generating equation for the phenotype, we examine properties of *estimated* PGS below. This model remains equivalent to equation (1) because subtracting $\bar{g}_{ij}$ terms, then summing genome-wide, ultimately subtracts a term linear in genome-wide ancestry which is absorbed into the α_i and μ^o terms. (Later, when we condition on this local ancestry to derive the ANCHOR approach, we will add local mean frequency terms that do impact behaviour.)

If the γ_j coefficients *do not* depend on genome-wide (or local) ancestry, then these coefficients will be identical for any group of admixed individuals (e.g. individuals binned by their genome-wide ancestry), and also will be shared with the coefficients of model (1) for non-admixed populations of the previous section. We will consider behaviour under this null model first. If there exist gene-gene or gene-environment effects that are correlated with ancestry, then the coefficients γ_j will vary among these groups. We will extend to this setting in section 0.9.

Note that this model applies across individuals with differing proportions θ_i of ancestry A (and other ancestries), but allows for linear effects of ancestry on the phenotype in the model, via the coefficients μ^o that are applied to the vector C_i of ancestries for individual i. Here, ϵ_i^o is the "true" (optimal) residual if we knew the model fully; ϵ_i^o is independent of the other random variables, with mean zero and finite variance σ^2; similarly μ^o etc represent the other true underlying parameters.

We next consider fitting the model in practice, now using *estimated* coefficients on each SNP. Introducing the indicator of ancestry A at SNP j, I_j, it is natural to consider fitting the model based on applying the separate models for A and E local ancestry from the previous section, for the relevant genome

pieces:

$$Y_i = \alpha + \beta_A \sum_{j=1}^{S} \hat{\gamma}_j I_j \left(g_{ij} - \bar{g}_j^A\right) + \beta_E \sum_{j=1}^{S} \hat{\gamma}_j (1 - I_j) \left(g_{ij} - \bar{g}_j^E\right) + \mu C_i + \epsilon_i. \quad (5)$$

If we define the ancestry A PGS

$$APGS = \sum_{j=1}^{S} \hat{\gamma}_j I_j \left(g_{ij} - \bar{g}_j^A\right)$$

(the estimated mean-centred PGS, summing only over those parts of the genome with ancestry A), and similarly for $EPGS$, then we can express this linear model as

$$Y \sim EPGS + APGS + coregressors.$$

Thus, this model can be fit from real data, provided $EPGS$ and $APGS$ can be obtained. We wish to use this model to approximate the true underlying model (1)/(4). Properties of the resulting coefficients β_A and β_E and their estimators are the key to the ANCHOR approach.

0.4 The impact of centring conditional on local ancestry

In the previous section, we form terms $EPGS$ and $APGS$ by mean-centring genotypes, and doing so *conditional* on the respective E and A ancestries of the segments of the genome used to construct these "European" and "African" polygenic scores. Note that $\bar{g}_j^A$ and $\bar{g}_j^E$ differ from each other as the mean frequencies in the respective populations, and also differ from the overall average $\bar{g}_{ij}$ (see above). In this section, we will call this "model I".

However, there are several natural alternative approaches, and it is not obvious yet which is better. First, one could simply use the non-centred genotypes,"model II":

$$APGS = \sum_{j=1}^{S} \hat{\gamma}_j I_j g_{ij}$$

$$EPGS = \sum_{j=1}^{S} \hat{\gamma}_j (1 - I_j) g_{ij}.$$

Alternatively, one could centre genotypes, but do so using the overall mean allele frequency, "model III":

$$APGS = \sum_{j=1}^{S} \hat{\gamma}_j I_j \left(g_{ij} - \bar{g}_j\right).$$

Finally one could centre based on the individual-specific mean allele frequency, "model IV":

$$APGS = \sum_{j=1}^{S} \hat{\gamma}_j I_j \left(g_{ij} - \bar{g}_{ij}\right).$$

For each approach, we could then fit the model

$$Y \sim EPGS + APGS + coregressors$$

as before. Note that models (II-IV) are actually all equivalent, yielding identical $EPGS$ and $APGS$ coefficients, which are the focus of our interest here - because relative to approach II, approaches III and IV add on terms respectively independent of, and linear in, genome-wide ancestry for each individual to the overall PGS, and these may be absorbed into the other regression coefficients. Thus we may focus on comparing model IV and the model I of (5), which *do* result in differing coefficients in general. Rewriting model IV to resemble the centred case, we will have:

$$\begin{aligned} Y_i =& \alpha + \beta_A \sum_{j=1}^{S} \hat{\gamma}_j I_j \left(g_{ij} - \bar{g}_j^A\right) + \beta_E \sum_{j=1}^{S} \hat{\gamma}_j (1 - I_j) \left(g_{ij} - \bar{g}_j^E\right) \\ &+ \sum_{j=1}^{S} \left[\pi_j I_j + \lambda_j (1 - I_j)\right] + \mu C_i + \epsilon_i. \end{aligned} \tag{6}$$

where $\pi_j = \beta_A \hat{\gamma}_j \left[\bar{g}_j^A - \bar{g}_{ij}\right]$ and $\lambda_j = \beta_E \hat{\gamma}_j \left[\bar{g}_j^E - \bar{g}_{ij}\right]$. Here the π_j, λ_j are non-independent coefficients. We will assume wlog the third sum has zero expectation, by otherwise simply subtracting the corresponding fraction c of the expectation from each π_j and λ_j term, and simply adjusting α without altering other parameters. Moreover by setting $\pi_j = \lambda_j = 0$ we obtain model I, so these coefficients capture the difference. Importantly, these terms involve the coefficients β_A and β_E, so non-identical coefficients will be obtained by fitting each model.

In the following section, we will use equation (6) to consider inference using models I and II, and hence I-IV, simultaneously. We will show that under the null of shared effects, model I yields values for β_A and β_E that do not vary among groups of varying proportions of ancestry from populations A and E, while this is not the case for model II. The derivation works by first showing that terms in (6) may be considered independently in terms of their least-squares contributions. As well as this theoretical result, we observe this effect in practice for our simulations (Extended Data Fig.9).

These results mean that centring conditional on local ancestry is important for our approach ANCHOR to work. (They also imply that improved phenotype prediction might occur for real data using such centring and then adjusting by local ancestry, and may have implications for existing approaches also, but we do not explore this further here.)

0.5 Expected squared residuals

Consider fitting the model (6) of the previous section, generalising equation (5), in the null setting where equation (4) is correct i.e. the true parameters γ_j are unchanging across individuals i. These provide two ways of writing Y_i, which must then agree (relating the two sets of residuals). In particular, individuals have varying genome-wide ancestry θ_i. Intuitively, one might expect that the mean coefficients of $EPGS$ and $APGS$ will equal their values β_E, β_A from section 0.2, and *not* depend on θ_i. If this is the case then equation (5) is well defined, in the sense that we obtain the same coefficients across distinct samples, regardless of their ancestry proportions - for example across samples binned according to θ_i. Below we show that under our generating model (4), this is the case, *provided* we use mean-centred scores as in (5). We note in passing that by our initial assumptions, conditional on genome-wide ancestry the terms $EPGS$ and $APGS$ are each uncorrelated with (in fact independent of) the coregressors (and uncorrelated with this ancestry itself), so in large samples the estimated coefficients for the coregressors are approximately independent of those for the PGS terms. Note also that the mean-centring works differently for the two ancestries A and E, due to different frequencies of mutations in each group.

Next, we will consider properties of the mean estimates of the parameters in equation (6) using least-squares estimation. From section 0.1, upon fitting Equation (6), the least-squares fit results in unbiased parameter estimates, whose expectations minimise the mean sum of squares of residuals, as we average across phenotype values Y and predictors X. It is then sufficient to consider this mean squared residual for a single individual i, with particular ancestry θ_i. In fact we may write using equations (4) and (6):

$$\begin{aligned}
&\alpha^o + \sum_{j=1}^{S} \gamma_j \left(g_{ij} - \bar{g}_{ij}\right) + \mu^o C_i + \epsilon_i^o \\
&= \alpha + \beta_A \sum_{j=1}^{S} \hat{\gamma}_j I_j \left(g_{ij} - \bar{g}_j^A\right) + \beta_E \sum_{j=1}^{S} \hat{\gamma}_j (1 - I_j) \left(g_{ij} - \bar{g}_j^E\right) \\
&\quad + \sum_{j=1}^{S} \left[\pi_j I_j + \lambda_j (1 - I_j)\right] + \mu C_i + \epsilon_i.
\end{aligned}$$

From above the expected values of least-square parameter estimates are those minimising $\mathbb{E}\left(\epsilon_i^2\right)$ (for any i). Solving for ϵ_i, note C_i is uncorrelated with the centred genotypes that appear in the other terms, which also have mean zero. Therefore the contribution from the non-β parameters to $\mathbb{E}\left(\epsilon_i^2\right)$ is simply additive, and vanishes if and only if $\alpha = \alpha^o$ and $\mu = \mu^o$, so these parameter values are unchanged relative to equation (4), i.e. our estimates for these parameters

are unbiased. Upon removing these vanishing terms

$$\sum_{j=1}^{S} \gamma_j \left(g_{ij} - \bar{g}_{ij}\right) + \epsilon_i^o = \beta_A \sum_{j=1}^{S} \hat{\gamma}_j I_j \left(g_{ij} - \bar{g}_j^A\right) + \beta_E \sum_{j=1}^{S} \hat{\gamma}_j (1 - I_j) \left(g_{ij} - \bar{g}_j^E\right)$$
$$+ \sum_{j=1}^{S} \left[\pi_j I_j + \lambda_j (1 - I_j)\right] + \epsilon_i.$$

A rearrangement yields

$$\begin{aligned}
\epsilon_i = & \sum_{j=1}^{S} \gamma_j \left(g_{ij} - \bar{g}_{ij}\right) - \beta_A \sum_{j=1}^{S} \hat{\gamma}_j I_j \left(g_{ij} - \bar{g}_j^A\right) - \beta_E \sum_{j=1}^{S} \hat{\gamma}_j (1 - I_j) \left(g_{ij} - \bar{g}_j^E\right) \\
& - \sum_{j=1}^{S} \left[\pi_j I_j + \lambda_j (1 - I_j)\right] + \epsilon_i^o \\
= & \left(\sum_{j=1}^{S} I_j \left[\gamma_j - \beta_A \hat{\gamma}_j\right] \left(g_{ij} - \bar{g}_j^A\right) \right) && \text{(I)} \\
& + \left(\sum_{j=1}^{S} (1 - I_j) \left[\gamma_j - \beta_E \hat{\gamma}_j\right] \left(g_{ij} - \bar{g}_j^E\right) \right) && \text{(II)} \\
& + \sum_{j=1}^{S} I_j \left(\gamma_j [\bar{g}_j^A - \bar{g}_{ij}] - \pi_j\right) + (1 - I_j) \left(\gamma_j [\bar{g}_j^E - \bar{g}_{ij}] - \lambda_j\right) && \text{(III)} \\
& + \epsilon_i^o. && \text{(IV)}
\end{aligned}$$

We consider the properties of the labelled specific terms on the RHS as we average over the genotype g_{ij} and ancestry I_j (each varying across sampled individuals). It will be convenient to write the overall genotype

$$g_{ij} = I_j g_{ij}^A + (1 - I_j) g_{ij}^E$$

where g_{ij}^A represents a random draw from the genome of a single ancestry A individual for each j, and similarly for g_{ij}^E. SNPs in different ancestry segments must come from different ancestors, so g_{ij}^A and g_{ij}^E are independent, and independent of I_j also. This allows us to regard g_{ij}^A as existing even if $I_j = 0$, since it does not impact the observed genotype, and similarly for g_{ij}^E: this will simplify taking expectations. In particular, within term (I) we may write $I_j(g_{ij} - \bar{g}_j^A) = I_j(g_{ij}^A - \bar{g}_j^A)$ which involves only independent random variables, and similarly for term (II).

Using this, term (I) and term (II) immediately have mean 0, and expected product 0, since for example $\mathbb{E}(g_{ij}^A) = \bar{g}_j^A$ and by independence of g_{ij}^A and g_{ij}^E. Moreover the expected products of term (III) and terms (I) and (II) are zero. To see this, first take the expectation of the product of e.g. term (I) and term (III) conditional on the value of I_j for each j. Term (III) is conditionally

constant, while terms (I) and (II) have mean zero after averaging over g_{ij}^A and g_{ij}^E. Thus the conditional expected product of term (I) and term (III) is zero, so the unconditional expectation is also. Finally, term (IV) is independent of the other terms so also has expected product with the other terms of zero. Thus, to calculate $\mathbb{E}\left(\epsilon_i^2\right)$ we must only sum the mean squares of terms (I)-(IV). The expectation of squared term (IV) is σ^2 and does not depend on the other parameters, so may be ignored in what follows. Now considering term (I):

$$
\begin{aligned}
&\mathbb{E}\left[\left(\sum_{j=1}^{S} I_j\left[\gamma_j-\beta_A\hat{\gamma}_j\right](g_{ij}^A-\bar{g}_j^A)\right)^2\right]\\
&=\sum_{j=1}^{S}\sum_{k=1}^{S}\left[\gamma_j-\beta_A\hat{\gamma}_j\right]\left[\gamma_k-\beta_A\hat{\gamma}_k\right]\mathbb{E}\left(I_jI_k(g_{ij}^A-\bar{g}_j^A)(g_{ik}^A-\bar{g}_k^A)\right)\\
&=\sum_{j=1}^{S}\sum_{k=1}^{S}\left[\gamma_j-\beta_A\hat{\gamma}_j\right]\left[\gamma_k-\beta_A\hat{\gamma}_k\right]\mathbb{E}\left(I_j(g_{ij}^A-\bar{g}_j^A)(g_{ik}^A-\bar{g}_k^A)\right)\\
&=\theta_i\sum_{j=1}^{S}\sum_{k=1}^{S}\left[\gamma_j-\beta_A\hat{\gamma}_j\right]\left[\gamma_k-\beta_A\hat{\gamma}_k\right]\mathbb{E}\left((g_{ij}^A-\bar{g}_j^A)(g_{ik}^A-\bar{g}_k^A)\right)\\
&=\theta_i\mathbb{E}\left[\left(\sum_{j=1}^{S}\left[\gamma_j-\beta_A\hat{\gamma}_j\right](g_{ij}^A-\bar{g}_j^A)\right)^2\right].
\end{aligned}
$$

Here the third line follows from our assumption that ancestry changes more slowly than LD, so that either $I_k = I_j$ or otherwise $\mathbb{E}\left((g_{ij}^A-\bar{g}_j^A)(g_{ik}^A-\bar{g}_k^A)\right)=0$ because SNPs k and j are not in LD. The final expression depends only on β_A among parameters to be fit. By inspection, the β_A value maximising this expression is unchanging with θ_i provided the γ_j terms remain fixed, because the distribution of g_{ik}^A is just that of genotypes of individuals from population A. When $\theta_i = 1$, the entire genome is from population A so our model reduces to the homogeneous case. Therefore the optimal β_A coefficient for this term is that for the non-admixed case where we simply regress the phenotype of a sample drawn from population A on the centred APGS.

By the same argument replacing ancestry A with ancestry E, term (II) has mean square which depends only on β_E and not on other parameters to be fit. It is minimised by choosing the same β_E coefficient for this term as for a non-admixed case, where we regress the phenotype of a sample drawn from population E on the centred EPGS. Finally, note that by construction term (III) has mean zero and depends only on I_j, the local ancestry. Then term (III) equals:

$$
\sum_{j=1}^{S}(1-\theta_i)I_j\left(\gamma_j[\bar{g}_j^A-\bar{g}_j^E]-\pi_j\right)-\theta_i(1-I_j)\left(\gamma_j[\bar{g}_j^A-\bar{g}_j^E]-\lambda_j\right)
$$

(simplifying further does not much aid interpretability). In principle, we could make this term vanish by setting $\pi_j = \lambda_j = \gamma_j[\bar{g}_j^A - \bar{g}_j^E]$, and this would be optimal. Unfortunately, this requires complete knowledge of the polygenic score which we do not possess! Further, we cannot easily estimate this term from GWAS data by conducting only a GWAS in a population similar to population E, for example a GWAS of UKB White British samples, if E represents European ancestry. That is because we do not know causal SNPs, preventing us from estimating their frequencies in population A accurately.

0.6 Estimates of β_A and β_E in the ANCHOR approach

In the ANCHOR approach, we use ancestry-conditional mean-centring, and this implies we set $\pi_j = \lambda_j = 0$ (see "The impact of centring conditional on local ancestry"). In this case, by inspection the expected mean square of term (III) has some value depending on properties of θ_i, but *not* on β_A or β_E. Therefore $\mathbb{E}\left(\epsilon_i^2\right)$ depends on these parameters only through terms (I), (II), involving β_A, β_B respectively. Thus, from above, $\mathbb{E}\left(\epsilon_i^2\right)$ is maximised by the same values of these coefficients as estimated from samples of individuals from populations A only and E only respectively. The "true" parameter values in the model then equal these values, which large-sample estimates converge to under our modelling assumptions (we describe variance estimation below). This is our main result: because we may estimate such coefficients in practice, testing them for equality with those estimated from non-admixed samples, or other admixed samples, represents a test of shared effect sizes (below, we extend to consider properties of these coefficients if effect sizes differ between groups).

Term (III) is stochastic (depending on local ancestry) and has mean zero, but non-zero mean square which depends on i through θ_i. This can be thought of as random noise, additional to the noise term ϵ^o in the phenotype. Therefore although this does not asymptotically bias parameter estimates, it will add additional noise in their estimation. This term almost vanishes in three useful cases: for individuals where only a small amount of ancestry is from population A, or from population E, or for individuals where one parent is entirely from population A and the other almost entirely from population E. In other cases, estimation of this term seems a useful topic for future research, and we note it contains information about genetic differences between populations A and E.

0.7 Biases using non-centred polygenic scores

As described above ("The impact of centring conditional on local ancestry"), approaches that do not use ancestry-conditional mean-centring are equivalent to setting $\pi_j = \beta_A \hat{\gamma}_j \left[\bar{g}_j^A - \bar{g}_{ij}\right] - c$ and setting $\lambda_j = \beta_E \hat{\gamma}_j \left[\bar{g}_j^E - \bar{g}_{ij}\right] - c$, where c is a constant chosen to yield zero mean for term (III). Critically, these involve the parameters β_A and β_E we seek to infer, so impact estimates of these parameters in principle. After a little simplification, the expected squared value of term

(III) in our equations above becomes

$$\mathrm{Var}\left(\sum_{j=1}^{S} I_j\left(\bar{g}_j^A - \bar{g}_j^E\right)\left(\gamma_j - \left[(1-\theta_i)\beta_A + \theta_i\beta_E\right]\hat{\gamma}_j\right)\right).$$

This term can be relatively large because it depends on ancestry variation, which can operate at broad genomic scales. Note that it is non-identifiable, and optimised whenever $(1-\theta_i)\beta_A + \theta_i\beta_E$ equals some value δ; in general this takes some arbitrary value. Our estimates for β_A and β_E using these non-centred polygenic scores must minimise the sum of terms (I-III), so e.g. the inferred value of β_A balances contributions of (I) and (III). Therefore we expect if θ_i is close to zero so most of the genome is from population E, term (II) will contribute strongly but (I) very weakly, so β_E will converge to its non-admixed value, while β_A will then converge towards some balance between δ and its non-admixed value. Conversely if θ_i is close to 1, β_E will converge in the direction of δ relative to its non-admixed value, and β_A towards its non-admixed value. Thus, we predict that in general, using non-centred polygenic scores will yield coefficient estimates that vary with genome-wide ancestry proportions, even in the case where no true differences in effect sizes exist. We observe this property in our simulated data, and see similar patterns suggesting this issue occurs in real data (Extended Data Fig.8 and Supplementary Fig.8-12).

There are a few cases where mean-centring has no effect: first, if I_j is constant along the genome, i.e. the same extremes mentioned in the previous section, including (as expected) the non-admixed cases. However, in practice we recommend masking uncertain or short ancestry segments so that the modelling assumptions of ANCHOR are approximately met (e.g. of larger segments than the extent of LD in the population). In this case the resulting random masking will effectively set the value $I_j = 0$ if marker j is masked and adds additional variation in term (III), predicted to contribute to the bias. Secondly, if the regression coefficients γ_j are perfectly estimated we obtain $\beta_A = \beta_E = 1$ as a solution, but we do not expect to be close to this case in practice unless our PGS includes only the strongest GWAS hits. In contrast, biasing due to this effect is greatest in settings where mis-estimated γ_j values occur at SNPs of strongly divergent frequencies in the two populations.

0.8 Parameter estimation in real samples and simulated data

In the UK Biobank (UKB) data, we analyse 8003 individuals whose ancestry is mainly derived from a mixture of sub-Saharan African ancestry (referred to henceforth as ancestry A), and European (especially British and Irish) ancestry (ancestry E). In practice, we run Hapmix to obtain realised versions of the haplotype-specific genotypes, jointly with the inferred ancestries I_{ij} at SNP j for individual i. Previous work (Price et al. 2009)[1] has shown that this yields highly accurate ancestry estimates. Analysing data for trios suggests as expected

that there is high accuracy of ancestry imputation in these data (Supplementary Fig.7) and by comparing to direct phasing, that the ancestry A and ancestry E polygenic scores are accurately estimated using Hapmix (Extended Data Fig.7), for a range of traits and across individuals.

To construct the $EPGS$ and $APGS$ terms, we use PGS β estimates constructed from the UKB analysis of individuals with British and Irish ancestry (Methods); note this population is closely related to group E. We also require the mean frequencies $\bar{g}_j^E$ and $\bar{g}_j^A$ of each SNP in the ancestral groups. We may obtain genome-wide ancestry estimates θ_i for each individual by taking the proportion of their genome inferred by Hapmix to come from population A (i.e. the African-like ancestry). Then noting that we may model

$$\mathbb{E}\left(g_{ij}\right) = \theta_i \bar{g}_j^A + (1-\theta_i)\bar{g}_j^E$$

we fit a linear regression of the observed genotypes g_{ij} on the left hand side against the values θ_i and use the resulting coefficients to estimate the two required means. Given the large sample size, these estimates are expected to closely match the true SNP frequencies for the specific admixing groups (they also correlate strongly with the frequencies of the same variants in 1000G cohort, Supplementary Fig.18).

Having obtained $EPGS$ and $APGS$, for different phenotypes and sets of individuals we first fit the model of equation (5), using our ACs, age, and gender as coregressors. We separately fitted models for each of 53 phenotypes (Main text; Figure 4-5) within UKB, binning individuals according to their genome-wide ancestry. This supplement demonstrates that if the PGS operates consistently (i.e. with conserved true effect sizes) across ancestries, then we estimate the same mean values for β_E and β_A asymptotically for each bin, and β_E will also approximately equal the value obtained directly for the large number of individuals within UKB of British and Irish ancestry, which we estimate as $\beta_{Obs.Eu}$. To obtain confidence intervals for the underlying parameter values, we used bootstrapping (1000 bootstrapped sample replicates). To reduce noise, we either combined all 8003 samples for a single trait (Figure 4-5: estimators β_{Eu}^{All} and β_{Af}^{All} for β_E, β_A and their bootstrapped confidence intervals are plotted) or compared bins using average values across all traits (Figure 4-5). We observe highly consistent estimates for β_E in all bins ($EPGS$ terms) and similarly consistent estimators for β_A ($APGS$), though these values are much lower.

To formally test whether β_A, β_E change with ancestry, indicating varying underlying effect sizes in admixed individuals, we fit a model allowing for an interaction of their effect sizes with genome-wide ancestry. Specifically, we generalised equation (5) to fit the model

$$Y_i = (\beta_A APGS + \beta_E EPGS)(1+\omega\theta_i) + coregressors$$

where the coregressors are as before. If $\omega = 0$ this reduces to the equation (5) case, but if $\omega \neq 0$ then this model allows the effect sizes γ_j of SNPs to be altered on average as mean ancestry varies. This could capture effects due to gene-gene interactions and/or gene-environment interactions, which correlate with

ancestry. We numerically obtain the least-squares estimates of the parameters of this model, and then evaluate confidence intervals via bootstraps as above. The $\omega = 0$ case corresponds to the null of no variation in underlying effect sizes with ancestry, and so we accept the null if zero lies within the confidence interval for ω. Although we perform a 2-sided test, note that if interactions do exist, we expect to observe $\hat{\omega} < 0$ in most cases when the GWAS producing the PGS was performed in population E or a close surrogate, for the same reason we expect $\beta_A < \beta_E$ in this case: the SNPs we identify will tend to have large (mean) effects in population E.

Note that $\beta_{Eu}^{Eu} = \hat{\beta}_E$ measures the estimated mean increase in the phenotype per unit increase in $EPGS$ for individuals with 100% population E ancestry, while $\beta_{Eu}^{Af} = \hat{\beta}_E(1 + \hat{\omega})$ can be interpreted as the corresponding term for individuals with (near) 100% population A ancestry; these terms, along with their corresponding $\hat{\beta}_A$ equivalents, are shown in Figure 4d,5, and ratios of terms are shown in Figure 5. If the 95% confidence interval for $\beta_{Eu}^{Af}/\beta_{Obs.Eu}$ includes 1, then there is no evidence for the underlying PGS coefficients varying between individuals of different ancestry, and this is true for almost all traits we analysed. Contrastingly, we see strong evidence for essentially all traits that $\beta_{Af}^{Af}/\beta_{Eu}^{Af} < 1$, so that differences in local LD and mutation frequencies impact all PGS examined, with our estimates for this ratio indicating a large disparity between groups.

Although Hapmix appears accurate in our testing, we also tested robustness of our estimates by masking uncertain or short ancestry segments (likely to be more prone to errors) from our estimation procedure and re-estimating coefficients; the results were virtually unchanged (Supplementary Fig.12). Errors in ancestry estimation or assignment of mutations to particular backgrounds would result in ancestry A mutations being assigned to E, and vice-versa. Therefore, we would expect such errors to result in more similar estimated coefficients for β_E and β_A than is truly the case, by mixing their contributions. Since we estimate coefficients for β_E that closely resemble those for non-admixed individuals from a population similar to E, rather than moving towards β_A, our results seem unlikely to be strongly impacted by such errors.

We analysed data for simulated phenotypes in exactly the same way as described above, i.e. matching the real data analyses. For simulated data (Extended Data Fig.8 and Supplementary Fig.8-9) and real data (Supplementary Fig.10-12) we also tested for bias when we applied the same approaches, but without performing local-ancestry-dependent mean-centring. The simulation data results show bias in the case of no centring, regardless of whether or not we perform masking base on ancestry segment size/uncertainty. The bias is stronger when we do perform masking - this last impact can be explained by the fact that masking, like ancestry segments, varies among individuals and so adds additional variable bias to the PGS (discussed in section 0.7).

0.9 Parameter estimation in real samples: variable effect sizes between populations

Consider the setting where population J is now an admixed population of interest, while we conduct an initial GWAS, and construct a PGS, in a population genetically identical to a second population I. Suppose these populations now have different effect sizes at SNPs, for example due to gene-gene or gene-environment interactions. For a particular causal SNP j with effect sizes γ_j^I and γ_j^J in each group, initially we assume these are normally distributed with mean zero and covariance matrix

$$\mathrm{Var}\begin{pmatrix} \gamma_j^I \\ \gamma_j^J \end{pmatrix} = \begin{pmatrix} \sigma^2 & \sigma^2\rho \\ \sigma^2\rho & \sigma^2 \end{pmatrix}$$

We previously considered the setting $\rho = 1$ (without imposing any normality assumption). We now consider properties of the estimators $\hat{\beta}_E$ and $\hat{\beta}_A$, if the two ancestries for population J are A and E, for any ρ. Conditional on the value of γ_j^I we have by properties of the Normal distribution that $\mathbb{E}\left(\gamma_j^J \mid \gamma_j^I\right) = \rho\gamma_j^I$. This is always reduced, because $\rho \leq 1$. Considering the estimators, we may still consider equations 4 and 6 and the subsequent derivation and properties of terms (I-IV) remain unchanged, if the true underlying PGS coefficient $\gamma_j = \gamma_j^J$ now represents the causal effect size of SNP j in population J. By normality we can represent $\gamma_j^J = \rho\gamma_j^I + \sqrt{1-\rho^2}\sigma Z_j$ where for each j, γ_j^I, the causal effect size in group I, and $Z_j \sim N(0,1)$, representing the perturbation of causal effects, are independent. From our earlier derivation, the large-sample estimator of β_E minimises term (II) and hence the following:

$$\mathbb{E}\left[\left(\sum_{j=1}^{S}\left[\gamma_j^J - \beta_E\hat{\gamma}_j\right](g_{ij}^E - \bar{g}_j^E)\right)^2\right]$$

$$= \mathbb{E}\left[\left(\sum_{j=1}^{S}\left[\rho\gamma_j^I + \sqrt{1-\rho^2}\sigma Z_j - \beta_E\hat{\gamma}_j\right](g_{ij}^E - \bar{g}_j^E)\right)^2\right]$$

$$= \rho^2\mathbb{E}\left[\left(\sum_{j=1}^{S}\left[\gamma_j^I - \frac{\beta_E}{\rho}\hat{\gamma}_j\right](g_{ij}^E - \bar{g}_j^E)\right)^2\right] + \sigma^2\left(1-\rho^2\right)\mathbb{E}\left[\left(\sum_{j=1}^{S} Z_j(g_{ij}^E - \bar{g}_j^E)\right)^2\right].$$

By inspection, the second term is independent of β_E while the first is identical after reparameterizing to the corresponding term for population I. If population I represents European ancestry, this is optimised by the parameter $\beta_{Obs.Eu}$. Hence the optimal value β_E is $\beta_E = \rho\beta_{Obs.Eu}$. Therefore, we can interpret the estimated relative coefficient $\hat{\beta}_E/\hat{\beta}_{Obs.Eu}$ as an estimate of the correlation in effect sizes, at causal SNPs, between European-ancestry and mixed African/European ancestry individuals (within any frequency bin).

We can relax the assumption of normality in this derivation, provided that the expectation of the true PGS in population J conditional on the true PGS

in population I *and* the estimated PGS in population I, is equal to ρ times the true PGS in population I. (This immediately holds under normality.) That is, ρ is the increase in mean trait value in the admixed group for a genetic perturbation causing an increase in mean trait value of 1 in population I, and the estimated PGS does not give information about population J not available from the *true* PGS in population I. We expect this second condition to normally hold in practice provided population I closely approximates the GWAS population used to produce the estimated PGS. We therefore define ρ in general to equal the limiting value of $\hat{\beta}_E/\hat{\beta}_{Obs.Eu}$. ρ equals the correlation in true genetic risks, if the variances in effect sizes are equal in I and J. In applications, even if effect size variances differ between two populations, the ratio $\hat{\beta}_E/\hat{\beta}_{Obs.Eu}$ estimates the mean increase in mean trait value in the admixed group for a genetic perturbation causing an increase in mean trait value of 1 in population E, which we note is a natural and useful interpretation of the meaning of the information captured by a PGS. In particular if the admixed group is more responsive to genetic perturbations overall it is possible to obtain $\rho > 1$, even in the presence of incomplete correlation in effect sizes between groups; note that this means the (true) PGS is more predictive of changes in the trait in the admixed group.

In our real analysis we never observe significant evidence of $\rho > 1$ for any trait (main text). Overall $\rho \approx 1$ explains our data well, and is very close to the average value across traits. Thus, we can conclude that an increase in a single unit in the "true" PGS (using European effect sizes) corresponds to an approximately unit increase in the average value of the trait in our UK Biobank African-ancestry individuals, also.

This is expected under a null model of largely shared (causal) effect sizes between groups and across traits. Under a model of differing causal effect sizes, observing $\rho \approx 1$ is possible in principle, but only if African-ancestry individuals are systematically more responsive to genetic perturbations (higher variance), across the relatively diverse group of traits we examined including molecular phenotypes as well as quantitative traits. This higher responsiveness would need to coincidentally cancel out the incomplete correlation in effect sizes. We are not aware of any literature evidence for such widespread and biased differential responses, and it would seem a non-parsimonious explanation compared to the simpler hypothesis of shared causal effect sizes. For this reason, we regard the data as strongly supportive that causal effect sizes are shared, whilst admitting the alternative theoretical possibility.

In our simulations incorporating either complete or incomplete correlation of effect sizes, we used the above covariance model $\gamma_j^J = \rho\gamma_j^I + \sqrt{1-\rho^2}\sigma Z_j$, simulated equal underlying variances and estimated ρ using $\hat{\beta}_E/\hat{\beta}_{Obs.Eu}$ to obtain values shown (Figure 4-5, Extended Data Fig.9 and Supplemenatry Fig.14).

References

[1] Alkes L Price, Arti Tandon, Nick Patterson, Kathleen C Barnes, Nicholas Rafaels, Ingo Ruczinski, Terri H Beaty, Rasika Mathias, David Reich, and

Simon Myers. Sensitive detection of chromosomal segments of distinct ancestry in admixed populations. *PLoS genetics*, 5(6):e1000519, 2009.
